# Effects of water quality, sanitation, handwashing, and nutritional interventions on diarrhoea and child growth in rural Kenya: a cluster-randomised controlled trial

**DOI:** 10.1016/S2214-109X(18)30005-6

**Published:** 2018-01-29

**Authors:** Clair Null, Christine P Stewart, Amy J Pickering, Holly N Dentz, Benjamin F Arnold, Charles D Arnold, Jade Benjamin-Chung, Thomas Clasen, Kathryn G Dewey, Lia C H Fernald, Alan E Hubbard, Patricia Kariger, Audrie Lin, Stephen P Luby, Andrew Mertens, Sammy M Njenga, Geoffrey Nyambane, Pavani K Ram, John M Colford

**Affiliations:** a Innovations for Poverty Action, Kakamega, Kenya; b Center for International Policy Research and Evaluation, Mathematica Policy Research, Washington, DC, USA; c Rollins School of Public Health, Emory University, Atlanta, GA, USA; d Department of Nutrition, University of California, Davis, CA, USA; e Department of Civil and Environmental Engineering, Stanford University, Stanford, CA, USA; f Department of Infectious Diseases and Geographic Medicine, Stanford University, Stanford, CA, USA; g Department of Civil and Environmental Engineering, Tufts University, Medford, MA, USA; h Division of Epidemiology, School of Public Health, University of California, Berkeley, CA, USA; i Division of Community Health Sciences, School of Public Health, University of California, Berkeley, CA, USA; j Division of Biostatistics, School of Public Health, University of California, Berkeley, CA, USA; k Eastern and Southern Africa Centre of International Parasite Control, Kenya Medical Research Institute, Nairobi, Kenya; l Department of Epidemiology and Environmental Health, School of Public Health and Health Professions, University at Buffalo, Buffalo, NY, USA

## Abstract

**Background:**

Poor nutrition and exposure to faecal contamination are associated with diarrhoea and growth faltering, both of which have long-term consequences for child health. We aimed to assess whether water, sanitation, handwashing, and nutrition interventions reduced diarrhoea or growth faltering.

**Methods:**

The WASH Benefits cluster-randomised trial enrolled pregnant women from villages in rural Kenya and evaluated outcomes at 1 year and 2 years of follow-up. Geographically-adjacent clusters were block-randomised to active control (household visits to measure mid-upper-arm circumference), passive control (data collection only), or compound-level interventions including household visits to promote target behaviours: drinking chlorinated water (water); safe sanitation consisting of disposing faeces in an improved latrine (sanitation); handwashing with soap (handwashing); combined water, sanitation, and handwashing; counselling on appropriate maternal, infant, and young child feeding plus small-quantity lipid-based nutrient supplements from 6–24 months (nutrition); and combined water, sanitation, handwashing, and nutrition. Primary outcomes were caregiver-reported diarrhoea in the past 7 days and length-for-age *Z* score at year 2 in index children born to the enrolled pregnant women. Masking was not possible for data collection, but analyses were masked. Analysis was by intention to treat. This trial is registered with ClinicalTrials.gov, number NCT01704105.

**Findings:**

Between Nov 27, 2012, and May 21, 2014, 8246 women in 702 clusters were enrolled and randomly assigned an intervention or control group. 1919 women were assigned to the active control group; 938 to passive control; 904 to water; 892 to sanitation; 917 to handwashing; 912 to combined water, sanitation, and handwashing; 843 to nutrition; and 921 to combined water, sanitation, handwashing, and nutrition. Data on diarrhoea at year 1 or year 2 were available for 6494 children and data on length-for-age *Z* score in year 2 were available for 6583 children (86% of living children were measured at year 2). Adherence indicators for sanitation, handwashing, and nutrition were more than 70% at year 1, handwashing fell to less than 25% at year 2, and for water was less than 45% at year 1 and less than 25% at year 2; combined groups were comparable to single groups. None of the interventions reduced diarrhoea prevalence compared with the active control. Compared with active control (length-for-age *Z* score −1·54) children in nutrition and combined water, sanitation, handwashing, and nutrition were taller by year 2 (mean difference 0·13 [95% CI 0·01–0·25] in the nutrition group; 0·16 [0·05–0·27] in the combined water, sanitation, handwashing, and nutrition group). The individual water, sanitation, and handwashing groups, and combined water, sanitation, and handwashing group had no effect on linear growth.

**Interpretation:**

Behaviour change messaging combined with technologically simple interventions such as water treatment, household sanitation upgrades from unimproved to improved latrines, and handwashing stations did not reduce childhood diarrhoea or improve growth, even when adherence was at least as high as has been achieved by other programmes. Counselling and supplementation in the nutrition group and combined water, sanitation, handwashing, and nutrition interventions led to small growth benefits, but there was no advantage to integrating water, sanitation, and handwashing with nutrition. The interventions might have been more efficacious with higher adherence or in an environment with lower baseline sanitation coverage, especially in this context of high diarrhoea prevalence.

**Funding:**

Bill & Melinda Gates Foundation, United States Agency for International Development.

## Introduction

An estimated 156 million children worldwide suffer from stunting (linear growth faltering) and are unlikely to reach their full potential as adults.^1^ Linear growth faltering is the most apparent sign of chronic undernutrition and is the physical manifestation of combined physiological and developmental insults. Early-life stunting leads to poor cognitive development in childhood, reduced economic productivity in adulthood, and increased risk of morbidity and mortality.^2^, ^3^ Because nutrient supplementation and counselling interventions for maternal, infant, and young child feeding have been only marginally successful at preventing growth faltering, exposure to faecal contamination in the environment has recently been hypothesised to lead to environmental enteric dysfunction, which features chronic immune stimulation and impaired nutrient absorption, thereby constraining a growth response to improved nutrition.^4^ In addition to the detrimental effects on growth and development, undernutrition was estimated to cause 45% of all child deaths in 2011, and it has long been recognised that undernutrition is an important determinant of susceptibility to infectious disease.^5^, ^6^ Diarrhoea is the second leading cause of death in children aged 1–59 months, contributing to almost 500 000 deaths in children younger than 5 years in 2015.^7^ Frequent diarrhoea is also associated with linear growth faltering.^8^ If there is a pathway independent of symptomatic diarrhoea linking environmental contamination to growth faltering, the benefits of improving water safety, sanitation, and handwashing could be underestimated because studies have generally focused on diarrhoea. It is unclear whether combined water, sanitation, handwashing, and nutritional interventions reduce diarrhoea or improve growth more than single interventions.

**Research in context**

**Evidence before this study**

Malnutrition and enteric infection are thought to act together to impair child health and survival, yet there is limited evidence of low cost, scalable interventions effective at breaking this cycle. A 2008 meta-analysis by Dewey and Adu-Afarwuah found that interventions offering nutrient supplementation or counselling on complementary feeding could result in modest improvements to child growth. Another meta-analysis by Waddington and Snilsveit in 2009 showed that water treatment or handwashing could prevent diarrhoea, but there had not been any randomised trials of the effect of sanitation on diarrhoea. During this study, five other randomised trials of the effects of sanitation on diarrhoea and growth were published, but three were limited by low adherence. Whether combining water, sanitation, handwashing, or nutrition interventions could result in added benefits for health and growth was not known.

**Added value of this study**

This trial is one of the first to provide experimental evidence on whether individual and combined water, sanitation, or handwashing interventions improve growth; combined water, sanitation, and handwashing interventions are more effective at reducing diarrhoea and growth faltering than any intervention alone; and nutrition counselling and supplementation are more effective when combined with improved water, sanitation, and handwashing. This is the first rigorous evaluation of upgrading from unimproved to improved latrines in sub-Saharan Africa. None of the interventions reduced diarrhoea, and only the interventions that included nutrition counselling and nutrient supplementation improved growth.

**Implications of all the available evidence**

Our results on growth effects are consistent with those from previous research on the combination of nutrition counselling and nutrient supplementation, finding modest effects on linear growth. It is possible that more intensive promotion and higher adherence would have resulted in larger effects, especially in this context of high diarrhoea prevalence, but few programmes are likely to be able to afford sustaining a more ambitious behaviour change programme than was included in this trial. In a context where most households already had an unimproved sanitation facility, provision of technologically simple interventions including chlorination for household treatment of drinking water, improved pit latrines, and handwashing stations—standard for most WASH programmes in rural areas of low-income countries—might not be sufficient to improve growth. By contrast with previous studies, this trial provided evidence that technologically simple water, sanitation, and handwashing interventions with adherence rates at least as high as most programmes achieve might not reduce childhood diarrhoea in all situations.

We aimed to investigate whether individual water, sanitation, handwashing, or nutrition interventions can reduce linear growth faltering; to assess whether combined water, sanitation, and handwashing interventions are more effective at reducing diarrhoea than individual interventions; and to investigate whether the combination of water, sanitation, handwashing, and nutrition interventions reduces growth faltering more than each individual intervention. A companion trial^9^ in Bangladesh evaluated the same objectives.

## Methods

### Study design

The Kenya WASH Benefits study was a cluster-randomised trial done in rural villages in Bungoma, Kakamega, and Vihiga counties in Kenya's western region (appendix p 11). We used a cluster design to facilitate the logistics of the behaviour change component of the interventions and minimise contamination between intervention and comparison households. We hypothesised that the interventions would improve the health of the index child in each household. We optimised the trial design to measure group-level differences in primary outcomes by including a large number of clusters, each comprising relatively few children (12 on average) with infrequent measurement. Each measurement round lasted roughly 1 year and was balanced across treatment groups and geography to minimise seasonal or geographic confounding when comparing outcomes across groups.

With active and passive control groups and six intervention groups (water; sanitation; handwashing; combined water, sanitation, and handwashing; nutrition; and combined water, sanitation, handwashing, and nutrition), the design enabled 11 comparisons of each intervention group with the active control; combined water, sanitation, and handwashing with each intervention alone; and combined water, sanitation, handwashing, and nutrition with nutrition alone, and combined water, sanitation, and handwashing. A double-sized active control group was used to increase power because there were six separate intervention comparisons against control.^10^ Households in the active control and all intervention groups were visited by community-based health promoters monthly to measure the child's mid-upper arm circumference. Health promoters did not visit households in passive control clusters. Measurement of outcomes, as well as water, sanitation, handwashing, and nutrition characteristics were measured in the passive control group at the same times as in other groups. The study design and rationale have been published previously.^10^

The study protocol was approved by the Committee for the Protection of Human Subjects at the University of California, Berkeley (protocol number 2011-09-3654), the institutional review board at Stanford University (IRB-23310), and the scientific and ethics review unit at the Kenya Medical Research Institute (protocol number SSC-2271). Under direction of the study investigators, Innovations for Poverty Action (IPA) was responsible for intervention delivery and data collection.

### Participants

Villages were eligible for selection into the study if they were rural, most of the population relied on communal water sources and had unimproved sanitation facilities, and there were no other ongoing water, sanitation, handwashing, or nutrition programmes. Participants were identified through a complete census of eligible villages. Within selected villages, women were eligible to participate if they reported that they were in their second or third trimester of pregnancy, planned to continue to live at their current residence for the next 2 years, and could speak Kiswahili, Luhya, or English well enough to respond to an interviewer administered survey. IPA staff formed clusters from one to three neighbouring villages to have six or more pregnant women per cluster after the enrolment survey. Outcomes were assessed in the children born from these pregnancies (index children), including twins. Although the study area is one of the areas with the highest HIV prevalence in Kenya, according to the 2012 Kenya AIDS Indicator Survey, the prevalence in women aged 15–64 years in the study area was below 6% (that survey did not include testing of children). Because there would not have been sufficient sample size to allow for subgroup analysis by HIV status, no attempt was made to identify participants who were HIV positive. Participants gave written informed consent before enrolment.

### Randomisation and masking

Clusters were randomly allocated to treatment using a random number generator with reproducible seed at the University of California, Berkeley. Groups of nine geographically-adjacent clusters were block-randomised into a double-sized active control; passive control; water; sanitation; handwashing; water, sanitation, and handwashing; nutrition; or water, sanitation, handwashing, and nutrition. Allocation by cluster identification number was communicated directly to the field team; investigators remained blinded to treatment assignments. Blinding of participants was not possible. Participants were informed of their treatment assignment after baseline data collection and might have known the treatment assignment of nearby villages. The health promoters and staff who delivered the interventions were not involved in data collection, but the data collection team could have inferred treatment status if they saw intervention materials in study communities.

### Procedures

The interventions were designed to maximise adherence to behaviours that could protect children from exposure to pathogens in their environment and improve diet quality. Formative research in the study area concluded that the health benefits of target behaviours were already well understood, but this knowledge was not sufficient to lead to action. As such, the behaviour change strategy and intervention materials were selected to create enabling environments, build supportive social norms, and target emotional drivers of decision making. The messages and delivery modes for the behaviour change strategy drew from existing information, education, and communication materials from organisations such as WHO, the Kenyan Government, UNICEF, and the Alive and Thrive network, and extensive previous qualitative work on the drivers of handwashing behaviours. Monthly visit modules were developed and pilot-tested to provide behavioural recommendations to mothers and other caregivers using key thematic constructs of convenience, nurturing care, and aspiration. We did a pilot randomised controlled trial^11^ to test the feasibility and acceptability of all the interventions and to collect data that allowed us to optimise the ratio of community-based promoters to study participants. To identify and correct systematic problems with adherence, staff confirmed that intervention materials were delivered to all study participants at the outset of the trial, and collected monitoring data on availability of intervention materials and recommended behaviours during unannounced visits to a random sample of at least 20% of participants in intervention groups 2, 6, 10, and 19 months after the interventions began.

Community-based promoters for intervention and active control groups were nominated by study mothers and other mothers of children younger than 3 years in the community. A second promoter was added if there were more than ten participants (single groups) or more than eight participants (combined groups) in the cluster, giving a total of 1031 promoters. Promoters attended 2 days (active control), 6 days (single groups), or 7 days (combined groups) of initial training led by study staff on how to measure mid-upper-arm circumference, communication skills, intervention-specific behaviour change messages and intervention materials, and the information they were expected to report to IPA. Refresher trainings were done 6, 12, and 18 months after the initial training. At 2, 4, 9, 15, and 21 months, study staff met with promoters in their clusters to observe visits and offer supportive supervision. Study staff called promoters monthly to collect information on their activities, intervention adherence in the households they visited, referrals to health centres, and births or deaths of study children. Promoters received a branded T-shirt, a mobile phone, job aids and intervention materials, and compensation of approximately US$15 per month for the first 6 months when they had more intensive engagement with the study participants, and $9 per month thereafter (the prevailing daily wage for unskilled labour in the study area is $1–2). Promoters were instructed to visit all participants in their cluster monthly and measure the child's arm circumference or the pregnant mother's abdomen.

In intervention groups, promoters engaged study participants and other compound members through interactive activities such as guided discussions using visual aids, song, and storytelling; resupplied consumable intervention materials; encouraged consistent practice of targeted behaviours; and helped troubleshoot barriers to adherence, including problems with intervention hardware and behavioural barriers. Promoters were provided with detailed plans for every visit, including key messages, scripts for discussing visual aids, and instructions for activities that emphasised the learning objectives. Visits lasted about 10 min in the active control group and 45–60 min in intervention groups during the first year when the key messages were conveyed. In the second year, promoters reinforced messages to maintain habits. All groups used messages on themes of nurture, aspiration, and self-efficacy, particularly in the context of a new birth. Interventions used convenience and social norms to encourage target behaviours.

In the three intervention groups that included water, promoters advocated treatment of drinking water with sodium hypochlorite. Chlorine dispensers for convenient water treatment at the point of collection were installed at an average of five communal water sources in the cluster and refilled as needed. Every 6 months, households in study compounds were given a 1 L bottle of chlorine for point-of-use water treatment in case households collected rainwater or used a source without a dispenser. Promoters used chlorine test strips during their regular visits to determine if the household was using chlorine, and negative results stimulated conversation about addressing barriers to chlorination.

In the three intervention groups that included sanitation, promoters advocated using latrines for defecation and safe disposal of children's and animals' faeces into a latrine. Existing unimproved latrines in study households were upgraded to improved latrines by installing a plastic slab, which also had a tight-fitting lid over the hole. New latrines were constructed for study households that did not have a latrine or whose latrine was unlikely to last for 2 years. All households in study compounds received a sani-scoop with a paddle as a dedicated faeces-removal tool. Finally, all households with children younger than 3 years in study compounds received plastic potties to facilitate toilet training and transfer of child faeces to the latrine.

In the three intervention groups that included handwashing, promoters advocated handwashing with soap before handling food and after defecation (including assisting a child). Study compounds were given two permanent, water-frugal handwashing stations intended to be installed near the food preparation area and the latrine. Handwashing stations were constructed of painted metal, with two foot-pedal-operated jerry-cans that dispensed a light flow of rinse water and soapy water. Promoters added chunks of bar soap to the soapy water container quarterly.

In the two intervention groups that included nutrition, a set of ten age-targeted modules were developed to enable promoters to advocate for best practices in maternal, infant, and young child feeding: recommendations for dietary diversity during pregnancy and lactation, early initiation of breastfeeding, exclusive breastfeeding until 6 months, introduction of appropriate and diverse complementary foods at 6 months, and continued breastfeeding through 24 months. Facilitators and barriers to behaviour change were elicited using formative research and health promoter guides were developed to address common barriers and questions. Study mothers with children between 6–24 months were provided with two 10 g sachets per day of a small quantity of lipid-based nutrient supplement (LNS; Nutriset; Malauny, France) that could be mixed into the child's food. LNS provided 118 kcal per day and 12 essential vitamins and ten minerals. Promoters explained that LNS was not to replace breastfeeding or complementary foods.

Promoters and intervention materials were introduced at community meetings roughly 6 weeks after enrolment. All interventions were delivered within 3 months of enrolment (appendix p 1). LNS was introduced to each child when they turned 6 months old. All handwashing stations and latrines were inspected within a month of construction, and a subset of households was periodically visited to observe group-specific indicators of intervention adherence. These data alerted study investigators to any issues with intervention implementation so they could be addressed consistently across all clusters and groups.

The enrolment survey included baseline demographics; assets; water, sanitation, and handwashing infrastructure; and target behaviours. Follow-up at 1 year and 2 years after intervention delivery consisted of an unannounced visit to study compounds to observe objective indicators of target behaviours (in all groups other than the passive control) and, on the following day, growth and health outcome measurements at a central location in the cluster (eg, a church or school).

Children identified as possibly malnourished (mid-upper-arm circumference <11·5 cm), either by the promoter during routine visits or by study staff during follow-up measurements, were referred to health facilities for treatment.

### Outcomes

Adherence to the interventions was assessed using objective, observable indicators where possible (appendix pp 2, 3). We calculated *Z* scores for length for age, weight for length, weight for age, and head circumference for age using the WHO 2006 child growth standards. All child deaths reported by the health promoters were confirmed by a staff nurse who visited households. All outcomes were prespecified. Primary outcomes were caregiver-reported diarrhoea in the past 7 days (based on all data from year 1 and year 2) and length-for-age *Z* score at year 2 in index children. Secondary and tertiary outcomes reported in this paper are length-for-age *Z* score at year 1; weight-for-length *Z* score, weight-for-age *Z* score, head circumference-for-age *Z* score at year 1 and year 2; prevalence of stunting (length-for-age *Z* score less than −2), severe stunting (length-for-age *Z* score less than −3), wasting (weight-for-length *Z* score less than −2), and underweight (weight-for-age *Z* score less than −2); and all-cause mortality. We excluded children from *Z*-score analyses if their measurements were outside biologically plausible ranges following WHO recommendations. More details on exclusion criteria, measurement protocols, and outcome definitions are in the appendix (p 1).

### Statistical analyses

Sample size calculations for the two primary outcomes were based on a minimum detectable effect of 0·15 in length-for-age *Z* score (intracluster correlation of 0·02 in our pilot study) and a relative risk of diarrhoea of 0·7 or smaller (assuming a 7-day prevalence of 12% in the active control group based on a pilot study to inform this trial) for a comparison of any intervention with the double-sized control group, assuming a type I error (α) of 0·05 and power (1–β) of 0·8, a one-sided test for a two-sample comparison of means, and 10% loss to follow-up.^10^, ^11^ Sample size calculations indicated 80 clusters per group, each with ten children.

Two biostatisticians, blinded to treatment assignment, independently replicated the analyses following the prespecified analysis plan with minor updates.^10^ We analysed participants according to their randomised assignment (intention to treat), regardless of adherence to the intervention, using the active control group as the comparator. We used paired *t* tests for unadjusted length-for-age *Z* score comparisons and the Mantel-Haenszel prevalence ratio and difference for unadjusted diarrhoea and stunting comparisons, with randomisation block defining matched pairs or stratification. In secondary analyses, we estimated prevalence ratios and differences, adjusting for baseline covariates using targeted maximum likelihood estimation.^12^ Analyses were done in *R* (version 3.2.3). We tested for the presence of between-cluster spillover effects using a non-parametric method described in the prespecified analysis plan, which tested whether primary outcomes were the same in control households with more versus fewer households receiving interventions within a 2 km radius. In an analysis that was not prespecified, we tested for intervention effects on diarrhoea using only year 1 data.

The trial is registered at ClinicalTrials.gov, number NCT01704105. IPA convened a data and safety monitoring board.

### Role of the funding source

The funders of the study approved the study design, but had no role in data collection, data analysis, data interpretation, or writing of the report. The corresponding author had full access to all the data in the study and had final responsibility for the decision to submit for publication.

## Results

**Figure 1:**
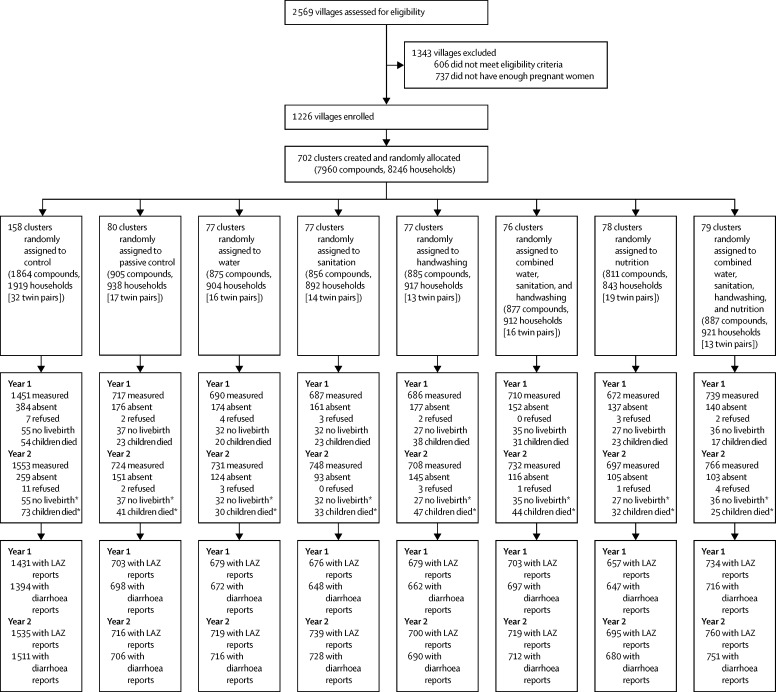
Trial profile and analysis populations for primary outcomes LAZ=length-for-age *Z* scores. *Stillbirth and child death counts are cumulative.

2569 villages were assessed for eligibility, of which 606 were excluded on the basis of village-level characteristics (primarily not meeting the study's rural criteria). 1226 villages were grouped into 702 clusters that had six or more pregnant women (figure 1). Between Nov 27, 2012, and May 21, 2014, 8246 pregnant women were enrolled in the study. 281 women did not have a livebirth and 140 women delivered twins. After at least three attempts to measure each child, 6659 (86%) of 7780 surviving children were measured at year 2, with diarrhoea reports for 6494 children and length-for-age *Z* score measures for 6583 children. Children were aged 2–18 months (median 12 months) at 1-year follow-up (January, 2014, to June, 2015) and aged 16–31 months (median 25 months) at 2-year follow-up (February, 2015, to July, 2016), but 11 184 (87%) of 12 841 children were in the target age ranges of 9–15 months at year 1 and 21–27 months at year 2 (appendix p 12).

Household characteristics were similar across groups at enrolment (table 1). Roughly three-quarters of participants collected drinking water from an improved source, but had to walk at least 10 min on average to the source. Over 80% of households owned a latrine, but less than 20% had access to an improved latrine. Less than 15% of households had soap available at a handwashing location. The prevalence of moderate-to-severe household hunger was 12% or lower.

**Table 1:** Baseline characteristics by intervention group

		**Active control (N=1919)**	**Passive Control (N=938)**	**Water (N=904)**	**Sanitation (N=892)**	**Handwashing (N=917)**	**Water, sanitation, and handwashing (N=912)**	**Nutrition (N=843)**	**Water, sanitation, handwashing, and nutrition (N=921)**
**Maternal**									
Age (years)		26 (6)	26 (7)	26 (6)	26 (7)	26 (6)	26 (6)	26 (6)	26 (6)
Completed at least primary education		916 (48%)	441 (47%)	447 (50%)	430 (48%)	402 (44%)	430 (47%)	409 (49%)	438 (48%)
Height (cm)		160 (6)	160 (7)	160 (6)	160 (6)	160 (6)	160 (6)	160 (7)	160 (7)
Study child is firstborn		490 (26%)	237 (25%)	205 (23%)	222 (25%)	208 (23%)	191 (21%)	206 (24%)	225 (25%)
**Paternal**									
Completed at least primary education		1098 (62%)	521 (60%)	532 (64%)	482 (58%)	500 (59%)	521 (61%)	491 (64%)	526 (62%)
Works in agriculture		749 (41%)	376 (43%)	378 (44%)	362 (43%)	363 (42%)	374 (43%)	343 (43%)	372 (43%)
**Household**									
Number of households per compound		2 (1)	2 (1)	2 (1)	2 (1)	2 (1)	2 (1)	2 (1)	2 (1)
Number of people per compound		8 (5)	8 (6)	8 (6)	8 (5)	8 (6)	8 (5)	8 (7)	8 (5)
Number of children <18 years in the household		3 (2)	3 (2)	3 (2)	3 (2)	3 (4)	3 (2)	3 (2)	3 (4)
Has electricity		122 (6%)	51 (5%)	60 (7%)	73 (8%)	67 (7%)	64 (7%)	58 (7%)	67 (7%)
Has a cement floor		107 (6%)	50 (5%)	71 (8%)	48 (5%)	41 (4%)	50 (5%)	48 (6%)	55 (6%)
Has an iron roof		1302 (68%)	600 (64%)	610 (68%)	587 (66%)	581 (63%)	574 (63%)	580 (69%)	615 (67%)
Owns a mobile phone		1526 (80%)	742 (79%)	705 (78%)	690 (77%)	722 (79%)	722 (79%)	685 (81%)	730 (79%)
Owns a motorcycle		185 (10%)	75 (8%)	81 (9%)	72 (8%)	91 (10%)	72 (8%)	81 (10%)	71 (8%)
**Drinking water**									
Primary drinking water source is improved*		1446 (76%)	699 (75%)	679 (75%)	675 (76%)	708 (78%)	624 (69%)	603 (72%)	697 (76%)
One-way walking time to primary water source (min)		11 (12)	12 (16)	12 (30)	10 (10)	11 (13)	11 (13)	11 (12)	11 (12)
Reported treating stored water		196 (13%)	92 (12%)	81 (11%)	94 (13%)	96 (13%)	97 (13%)	79 (12%)	106 (14%)
**Sanitation**									
Always or usually use primary toilet for defecation									
	Men	1778 (95%)	867 (95%)	828 (94%)	810 (94%)	845 (95%)	851 (95%)	785 (95%)	854 (95%)
	Women	1822 (96%)	898 (96%)	868 (96%)	840 (94%)	871 (96%)	877 (96%)	812 (96%)	872 (95%)
Daily defecating in the open									
	Children aged 3 to <8 years	145 (12%)	87 (14%)	74 (13%)	68 (13%)	81 (14%)	75 (13%)	82 (15%)	75 (12%)
	Children aged 0 to <3 years	789 (78%)	378 (77%)	376 (80%)	370 (75%)	358 (76%)	394 (77%)	363 (79%)	388 (78%)
Latrine									
	Own any latrine	1561 (82%)	774 (83%)	750 (83%)	722 (81%)	756 (83%)	754 (83%)	701 (83%)	764 (83%)
	Access to improved latrine	309 (17%)	153 (17%)	150 (18%)	131 (16%)	157 (19%)	153 (18%)	119 (15%)	143 (16%)
Human faeces observed in the compound		163 (9%)	79 (8%)	66 (7%)	72 (8%)	84 (9%)	73 (8%)	73 (9%)	87 (9%)
**Handwashing location**									
Has water within 2 m of handwashing location		487 (25%)	236 (25%)	242 (27%)	245 (28%)	245 (27%)	251 (28%)	228 (27%)	249 (27%)
Has soap within 2 m of handwashing location		164 (9%)	94 (10%)	91 (10%)	75 (8%)	83 (9%)	115 (13%)	90 (11%)	87 (9%)
**Food security**									
Prevalence of moderate-to-severe household hunger†		203 (11%)	113 (12%)	106 (12%)	91 (10%)	92 (10%)	101 (11%)	98 (12%)	104 (11%)

Data are n (%) or mean (SD). Percentages were calculated from smaller denominators than those shown at the top of the table for all variables because of missing values.

* Defined by WHO UNICEF Joint Monitoring Program's definition for an improved water source.

† Assessed by the Household Food Insecurity Access Scale.

Around 75% of households were visited by their promoter within the past month at year 1, but frequency of contact fell by year 2, with 40% or fewer households reporting a visit in the past month in each group (monitoring data suggest that most households were still visited at least every other month during the second year of the trial; see details in the appendix p 2, and table 2). Slightly less than half of households had detectable free chlorine in stored drinking water in the water group. Around 40% of drinking water samples tested in the water, sanitation, handwashing, and nutrition group had detectable free chlorine at year 1, which fell to around 20% by year 2. A high proportion of households (75%) had improved latrine access, which remained stable in year 1 and year 2 in households in the sanitation groups, increasing by more than 50% compared with the active control group. Reported safe disposal of children's faeces into a latrine fell by roughly half in all groups between year 1 and year 2, although the practice remained over twice as likely in the groups that included sanitation compared with other groups at year 1 and year 2. More than 75% of households in the intervention groups that included handwashing had water and soap available at a handwashing location at year 1, but this indicator also fell to about 20% by year 2. Adherence to LNS recommendations was high (≥95%) at year 1 and year 2, with children consuming a few more LNS sachets per month on average than would be expected at year 2. Across all indicators, adherence was comparable between the water, sanitation, and handwashing group and the water, sanitation, handwashing, and nutrition group compared with single intervention groups.

**Table 2:** Measures of intervention adherence by study group at enrolment, 1-year follow-up, and 2-year follow-up

	**Active Control (N=1919)**	**Passive Control (N=938)**	**Water (N=904)**	**Sanitation (N=892)**	**Handwashing (N=917)**	**Water, sanitation, and handwashing (N=912)**	**Nutrition (N=843)**	**Water, sanitation, handwashing, and nutrition (N=921)**
**Number of compounds assessed**								
Enrolment	1913/1919 (100%)	936/938 (100%)	902/904 (100%)	890/892 (100%)	914/917 (100%)	912/912 (100%)	843/843 (100%)	918/921 (100%)
Year 1	1043/1919 (54%)	..	477/904 (53%)	473/892 (53%)	501/917 (55%)	536/912 (59%)	454/843 (54%)	493/921 (54%)
Year 2	1458/1919 (76%)	..	696/904 (77%)	712/892 (80%)	690/917 (75%)	675/912 (74%)	650/843 (77%)	735/921 (100%)
**Visited by promoter in past month**								
Enrolment	..	..	..	..	..	..	..	..
Year 1	666/980 (68%)	..	338/445 (76%)	333/445 (75%)	333/480 (69%)	386/512 (75%)	344/433 (79%)	388/474 (82%)
Year 2	492/1412 (35%)	..	255/680 (37%)	278/692 (40%)	228/678 (34%)	241/649 (37%)	251/635 (40%)	259/710 (36%)
**Stored drinking water has detectable free chlorine**								
Enrolment	44/1529 (3%)	24/736 (3%)	20/720 (3%)	20/715 (3%)	30/743 (4%)	29/711 (4%)	14/661 (2%)	26/729 (4%)
Year 1	25/847 (3%)	..	151/385 (39%)	18/367 (5%)	20/417 (5%)	180/424 (42%)	9/392 (2%)	156/367 (43%)
Year 2	38/1365 (3%)	..	144/637 (23%)	17/641 (3%)	16/648 (2%)	112/598 (19%)	15/614 (2%)	128/652 (20%)
**Access to improved latrine**								
Enrolment	309/1788 (17%)	153/878 (17%)	150/844 (18%)	131/836 (16%)	157/847 (19%)	153/867 (18%)	119/794 (15%)	143/872 (16%)
Year 1	178/993 (18%)	..	74/461 (16%)	409/458 (89%)	65/486 (13%)	472/526 (90%)	63/424 (15%)	425/477 (89%)
Year 2	271/1381 (20%)	..	128/664 (19%)	534/683 (78%)	119/654 (18%)	529/644 (82%)	99/613 (16%)	561/706 (79%)
**Child faeces safely disposed of**								
Enrolment	114/721 (16%)	51/323 (16%)	53/310 (17%)	67/347 (19%)	54/319 (17%)	65/369 (18%)	33/310 (11%)	56/353 (16%)
Year 1	338/903 (37%)	..	158/424 (37%)	317/412 (77%)	157/431 (36%)	326/463 (70%)	155/391 (40%)	287/432 (66%)
Year 2	136/1320 (10%)	..	52/625 (8%)	240/643 (37%)	62/616 (10%)	205/597 (34%)	52/578 (9%)	219/657 (33%)
**Handwashing location has water and soap**								
Enrolment	96/1913 (5%)	58/936 (6%)	56/902 (6%)	42/890 (5%)	52/914 (6%)	64/912 (7%)	57/843 (7%)	53/918 (6%)
Year 1	124/1043 (12%)	..	53/477 (11%)	49/473 (10%)	381/501 (76%)	416/536 (78%)	61/454 (13%)	381/493 (77%)
Year 2	127/1458 (9%)	..	49/696 (7%)	57/712 (8%)	159/690 (23%)	130/675 (19%)	76/650 (12%)	152/735 (21%)
LNS sachets consumed (% expected)*								
Enrolment	..	..	..	..	..	..	..	..
Year 1	..	..	..	..	..	..	5264/5558 (95%)	5583/5838 (96%)
Year 2	..	..	..	..	..	..	3577/3136 (114%)	4028/3458 (116%)

Data are n (%), or %. Free chlorine in drinking water and LNS consumption were not measured at enrolment and were only measured in a subset of groups. LNS=lipid-based nutrient supplement.

* LNS adherence measured as reported proportion of 14 sachets consumed in the past week in index children aged 6–24 months.

**Figure 2:**
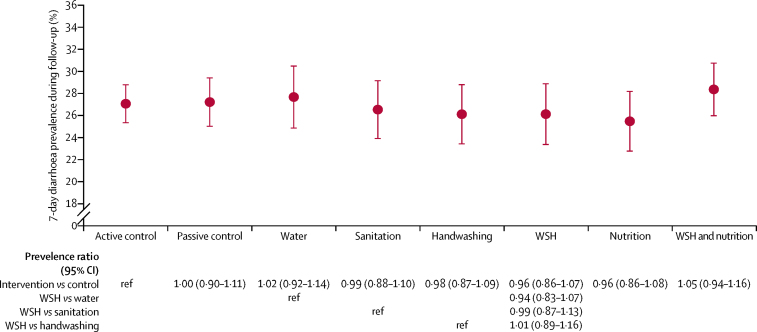
Intervention effects on diarrhoea prevalence 1 and 2 years after intervention Data are mean (95% CI). ref=reference. WSH=water, sanitation, and handwashing.

Diarrhoea prevalence over the past 7 days (combining data from year 1 and year 2) was 27·1% in children in the active control group (figure 2, table 3). The intracluster correlation for diarrhoea was 0·012. Compared with the active control group, the diarrhoea prevalence ratios across all groups were not significantly different from one and differences were not significantly different from zero (figure 2, table 3). Diarrhoea prevalence was the same in the combined water, sanitation, and handwashing group and the individual water, sanitation, and handwashing groups. Although adherence to the water and handwashing interventions was higher in year 1 than in year 2, in an analysis that was not prespecified, diarrhoea prevalence was not significantly lower in any of the intervention groups at year 1 (appendix p 12). The high diarrhoea prevalence was fairly stable over 2 years of follow-up and there were no apparent seasonal trends (appendix p 13). Although we had prespecified a sensitivity analysis by age group of child at year 2, we did not complete this analysis because sample sizes in the age group strata were smaller than expected.

**Table 3:** Diarrhoea prevalence from 1 and 2 years (combined) after intervention

	Mean*prevalence	**Unadjusted**†**prevalence difference (95% CI)**	**Adjusted**‡**prevalence difference (95% CI)**
**Intervention *vs* active control**			
Active control	27·1%	..	..
Passive control	27·2%	−0·0 (−2·9 to 2·9)	−0·4 (−3·3 to 2·4)
Water	27·7%	0·7 (−2·3 to 3·6)	0·4 (−3·2 to 4·0)
Sanitation	26·5%	−0·3 (−3·3 to 2·6)	−0·3 (−3·2 to 2·6)
Handwashing	26·1%	−0·6 (−3·5 to 2·3)	−1·1 (−4·0 to 1·8)
Water, sanitation, and handwashing	26·1%	−1·2 (−4·1 to 1·7)	−1·1 (−4·3 to 2·0)
Nutrition	25·5%	−1·0 (−4·0 to 2·0)	−0·6 (−4·0 to 2·7)
Water, sanitation, handwashing, and nutrition	28·4%	1·2 (−1·7 to 4·1)	0·7 (−2·4 to 3·7)
**Water, sanitation, and handwashing *vs* single groups**			
Water, sanitation, and handwashing	26·1%	..	..
Water	27·7%	−1·6 (−5·1 to 1·9)	−2·1 (−6·0 to 1·8)
Sanitation	26·5%	−0·2 (−3·6 to 3·2)	−0·8 (−4·5 to 2·9)
Handwashing	26·1%	0·4 (−3·2 to 3·9)	0·5 (−3·6 to 4·5)

* Post-intervention measurements in years 1 and 2 combined.

† Unadjusted estimates were estimated using a pair-matched Mantel-Haenszel analysis.

‡ Adjusted for prespecified covariates using targeted maximum likelihood estimation with data-adaptive model selection: field staff who collected data, month of measurement, household food insecurity, child age, child sex, mother's age, mother's height, mothers education level, number of children <18 years in the household, number of individuals living in the compound, distance in minutes to the primary water source, household roof, floor, wall materials, and household assets.

**Figure 3:**
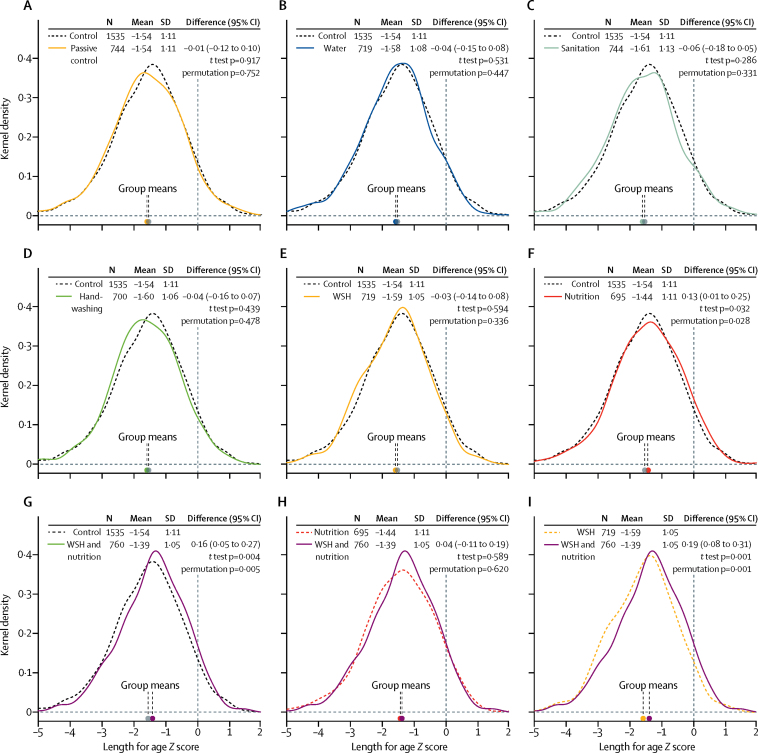
Intervention effects on length-for-age Z scores in 6583 children after 2 years of intervention Kernel density plots show the distribution of length-for-age *Z* scores; dashed lines are the comparison group distribution and solid lines are the active comparator distribution. (A) Passive control *vs* active control. (B) Water *vs* active control. (C) Sanitation vs active control. (D) Handwashing *vs* active control. (E) WSH *vs* active control. (F) Nutrition *vs* active control. (G) WSH and nutrition *vs* active control. (H) WSH and nutrition *vs* nutrition. (I) WSH and nutrition *vs* WSH. p values for *t* test are for differences in group means from zero; permutation p values test the null hypothesis of no difference between groups using a Wilcoxon signed-rank test statistic. WSH=water, sanitation, and handwashing.

By year 2, when children were between 16 and 31 months old (median 25 months), mean length-for-age *Z* score in children in the active control group was −1·54 (SD 1·11; figure 3). The intracluster correlation for length-for-age *Z* score was 0·037. Compared with the active control group, only nutrition and combined water, sanitation, handwashing, and nutrition had higher length-for-age *Z* score (mean difference in score 0·13 [95% CI 0·01–0·25] for nutrition; 0·16 [0·05–0·27] for combined water, sanitation, handwashing, and nutrition; figure 3). Children in the combined water, sanitation, handwashing, and nutrition group were not significantly taller than children in the nutrition group (mean difference 0·04 [95% CI −0·11 to 0·19]; figure 3). Most length-for-age *Z* score gains in these two groups were already apparent by year 1 (0·11 [–0·01 to 0·22] for nutrition; 0·12 [0·01–0·22] for combined water, sanitation, handwashing, and nutrition; appendix p 14).

Mean weight-for-age *Z* score at year 2 was higher in children in the nutrition and combined water, sanitation, handwashing, and nutrition groups than the mean of −0·72 (SD 1·01) in the active control group (table 4). Children in the active control group were close to WHO standards for weight-for-length *Z* score; however, weight-for-length *Z* score at year 2 was higher in the combined water, sanitation, handwashing, and nutrition group (table 4). There were no differences in mean head circumference for age *Z* score at year 2 between children in any of the intervention groups and those in the active control group. Results were similar at year 1, with the exception that differences in mean weight-for-length *Z* score between the active control and two groups with the nutrition intervention appear to have been numerically larger at year 1 (appendix p 15).

**Table 4:** Child growth *Z* scores at 2-year follow-up

	**N**	**Mean (SD)**	**Difference *vs* active control (95% CI)**	**Difference *vs* nutrition (95% CI)**	**Difference *vs* water, sanitation, and handwashing (95% CI)**
**Weight-for-age *Z* score**					
Active control	1548	−0·72 (1·01)	..	..	..
Passive control	721	−0·76 (0·97)	−0·04 (−0·13 to 0·05)	..	..
Water	727	−0·73 (1·00)	0·00 (−0·10 to 0·10)	..	..
Sanitation	747	−0·80 (1·05)	−0·07 (−0·19 to 0·04)	..	..
Handwashing	706	−0·77 (1·01)	−0·05 (−0·15 to 0·05)	..	..
Water, sanitation, and handwashing	725	−0·77 (0·98)	−0·02 (−0·12 to 0·08)	..	..
Nutrition	698	−0·65 (0·98)	0·11 (0·00 to 0·21)	..	..
Water, sanitation, handwashing, and nutrition	765	−0·60 (0·96)	0·14 (0·04 to 0·25)	0·04 (−0·07 to 0·15)	0·17 (0·05 to 0·30)
**Weight-for-length *Z* score**					
Active control	1536	0·11 (0·94)	..	..	..
Passive control	717	0·08 (0·92)	−0·04 (−0·13 to 0·05)	..	..
Water	719	0·14 (0·95)	0·04 (−0·06 to 0·13)	..	..
Sanitation	740	0·05 (0·97)	−0·05 (−0·14 to 0·05)	..	..
Handwashing	700	0·09 (0·93)	−0·02 (−0·11 to 0·06)	..	..
Water, sanitation, and handwashing	714	0·08 (0·92)	−0·02 (−0·10 to 0·07)	..	..
Nutrition	695	0·14 (0·92)	0·04 (−0·05 to 0·14)	..	..
Water, sanitation, handwashing, and nutrition	762	0·18 (0·90)	0·09 (0·00 to 0·19)	0·04 (−0·05 to 0·13)	0·12 (0·00 to 0·23)
**Head circumference-for-age *Z* score**					
Active control	1545	−0·27 (1·02)	..	..	..
Passive control	719	−0·27 (1·05)	0·00 (−0·10 to 0·10)	..	..
Water	727	−0·27 (1·03)	0·02 (−0·08 to 0·12)	..	..
Sanitation	745	−0·27 (1·04)	0·01 (−0·09 to 0·11)	..	..
Handwashing	705	−0·29 (0·99)	0·00 (−0·10 to 0·10)	..	..
Water, sanitation, and handwashing	729	−0·30 (0·96)	−0·03 (−0·12 to 0·06)	..	..
Nutrition	695	−0·23 (0·99)	0·05 (−0·05 to 0·15)	..	..
Water, sanitation, handwashing, and nutrition	763	−0·22 (0·99)	0·05 (−0·04 to 0·15)	−0·02 (−0·14 to 0·10)	0·08 (−0·05 to 0·20)

Median child age at 2-year follow-up was 2·05 years (IQR 1·93–2·16). All three secondary outcomes were prespecified.

Compared with the active control group, a smaller proportion of children in the combined water, sanitation, handwashing, and nutrition group were stunted (too short for their age; −5·4 percentage points [95% CI −9·4 to −1·4]), severely stunted (−2·7 percentage points [–5·1 to −0·2]), or underweight (−3·0 percentage points [–5·4 to −0·6]; table 5); no other groups appeared to affect these outcomes. Notably, there were no significant differences between the combined water, sanitation, handwashing, and nutrition and nutrition groups for any growth outcomes. 1% of active control children were wasted and the proportions were similar across all groups.

**Table 5:** Proportion of children stunted, severely stunted, wasted, and underweight at 2-year follow-up

	**n/N (%)**	**Difference *vs* active control (95% CI)**	**Difference *vs* nutrition (95% CI)**	**Difference *vs* water, sanitation, and handwashing (95% CI)**
Stunting*				
Active control	483/1535 (31%)	..	..	..
Passive control	223/716 (31%)	−1·7 (−5·9 to 2·5)	..	..
Water	233/719 (32%)	0·1 (−4·2 to 4·3)	..	..
Sanitation	255/739 (35%)	2·3 (−2·0 to 6·6)	..	..
Handwashing	235/700 (34%)	0·8 (−3·5 to 5·1)	..	..
Water, sanitation, and handwashing	236/719 (33%)	1·3 (−3·0 to 5·6)	..	..
Nutrition	201/695 (29%)	−3·2 (−7·5 to 1·1)	..	..
Water, sanitation, handwashing, and nutrition	203/760 (27%)	−5·4 (−9·4 to −1·4)	−2·3 (−7·1 to 2·5)	−5·8 (−10·6 to −1·0)
**Severe stunting**†				
Active control	143/1535 (9%)	..	..	..
Passive control	62/716 (9%)	−0·8 (−3·3 to 1·8)	..	..
Water	69/719 (10%)	−0·5 (−3·2 to 2·2)	..	..
Sanitation	77/739 (10%)	1·0 (−1·8 to 3·7)	..	..
Handwashing	59/700 (8%)	−1·1 (−3·7 to 1·5)	..	..
Water, sanitation, and handwashing	65/719 (9%)	0·2 (−2·4 to 2·8)	..	..
Nutrition	55/695 (8%)	−1·6 (−4·2 to 1·0)	..	..
Water, sanitation, handwashing, and nutrition	55/760 (7%)	−2·7 (−5·1 to −0·2)	−0·9 (−3·7 to 2·0)	−2·7 (−5·6 to 0·2)
**Wasting**†				
Active control	22/1536 (1%)	..	..	..
Passive control	10/717 (1%)	0·0 (−1·1 to 1·1)	..	..
Water	9/719 (1%)	−0·2 (−1·3 to 0·8)	..	..
Sanitation	19/740 (3%)	1·1 (−0·3 to 2·4)	..	..
Handwashing	6/700 (1%)	−0·5 (−1·5 to 0·4)	..	..
Water, sanitation, and handwashing	10/714 (1%)	0·2 (−0·9 to 1·2)	..	..
Nutrition	8/695 (1%)	−0·3 (−1·3 to 0·8)	..	..
Water, sanitation, handwashing, and nutrition	11/762 (1%)	−0·1 (−1·2 to 1·0)	0·2 (−1·0 to 1·4)	0·0 (−1·2 to 1·1)
**Underweight**†				
Active control	148/1548 (10%)	..	..	..
Passive control	70/721 (10%)	−0·4 (−3·0 to 2·2)	..	..
Water	76/727 (10%)	−0·1 (−2·8 to 2·7)	..	..
Sanitation	87/747 (12%)	1·6 (−1·2 to 4·4)	..	..
Handwashing	71/706 (10%)	0·5 (−2·2 to 3·3)	..	..
Water, sanitation, and handwashing	72/725 (10%)	0·5 (−2·3 to 3·2)	..	..
Nutrition	59/698 (8%)	−1·2 (−3·9 to 1·5)	..	..
Water, sanitation, handwashing, and nutrition	52/765 (7%)	−3·0 (−5·4 to −0·6)	−1·8 (−4·7 to 1·1)	−3·3 (−6·2 to −0·5)

Median child age at 2-year follow-up was 2·05 years (IQR 1·93–2·16).

* Prespecified secondary outcome.

† Prespecified tertiary outcome.

Differences in growth outcomes between the active control and intervention groups were similar in magnitude and precision when estimated using adjusted models (appendix pp 16–19). We found no evidence of between-cluster spillover effects (appendix p 20).

The cumulative incidence of all-cause mortality was 3·9% in the active control and ranged from 5·3% in the handwashing group to 2·8% in the combined water, sanitation, handwashing, and nutrition group; none of the differences between intervention groups and the active control were statistically significant at α=0·05 (figure 1, appendix p 21).

## Discussion

In the WASH Benefits cluster-randomised controlled trial, we found no effect of any interventions (improved water quality, safe sanitation, handwashing, nutrition, or combinations of the interventions) on caregiver-reported diarrhoea prevalence during the first 2 years of life, and improvements in growth were only observed in groups including the nutrition intervention (maternal, infant, and young child feeding counselling and LNS distribution). With a large sample size and high-quality anthropometric measurements, this trial was powered to detect small effects in diarrhoea prevalence and length-for-age *Z* score had they been present. Lower adherence to the water and handwashing interventions by the end of the 2 years of intervention does not seem to be the only explanation for the absence of benefits: there were also no reductions in diarrhoea or improvements in growth in children in the water, handwashing, sanitation, or combined water, sanitation, and handwashing groups even in the first year (a typical measurement point in previous trials), when community-based promoters were most active and adherence was higher, whereas almost all of the growth benefits in the nutrition group and combined water, sanitation, handwashing, and nutrition group were already manifest in the first year. Adherence to the interventions was comparable to or better than what a government or large non-governmental organisation might hope to achieve at scale (appendix p 22), with increases in adherence indicators of 30 percentage points or higher in all intervention groups relative to the control in the first year.

These findings contrast with several systematic reviews^13^, ^14^, ^15^ that have found significant protective benefits of water, sanitation, and hygiene interventions (including handwashing) on diarrhoea in efficacy trials, although most of these studies were shorter and had higher adherence. Results from other trials^16^, ^17^, ^18^ also showed no effect of improved sanitation on diarrhoea, although differences in contexts and interventions complicate comparisons between these trials. Our trial differed from previous trials in that the intervention shifted households from unimproved sanitation (rather than open defecation) to improved sanitation. Additionally, the prevalence of diarrhoea in this study population was high, consistent with prevalence in 12–23-month-old infants measured in the 2014 Kenya Demographic and Health Survey.^19^

A systematic review and meta-analysis^20^ of the effects of water quality and supply, sanitation, and hygiene interventions to improve growth identified only five randomised controlled trials of water or handwashing interventions, which did not suggest strong effects on growth, perhaps in part because the interventions lasted only 9–12 months. Since then, five more randomised trials of sanitation interventions have generated mixed evidence on child growth effects: two trials done in India and one in Indonesia had low adherence and no effect, and two done in settings with high rates of open defecation in India and Mali showed improvements in length-for-age *Z* score of 0·18–0·40 in children younger than 5 years.^16^, ^17^, ^18^, ^20^, ^21^, ^22^ The sanitation intervention in our trial was aligned with the focus on improved latrines initiated under the Millennium Development Goals, and the Sustainable Development Goals' recognition that children's faeces also need to be safely disposed of. This trial and its companion trial^9^ in Bangladesh suggest that a compound-level approach to upgrading existing latrines and safely disposing of children's faeces is not sufficient to improve child growth, and neither are water and handwashing interventions.

Conversely, counselling and LNS provided in the nutrition group improved length-for-age *Z* score by year 2. Compared with randomised controlled trials of LNS during complementary feeding, our finding of length-for-age *Z* score improvements of 0·13–0·16 in the nutrition groups falls in the middle of the spectrum between four trials: one from Malawi^23^ that reported no effect on length-for-age *Z* score, one from Haiti^24^ and one from Bangladesh^25^ that reported an effect on length-for-age *Z* score comparable to this study, and one from Burkina Faso^26^ that reported a larger effect on length-for-age *Z* score. Thus, there appears to be consistent evidence that LNS distribution together with some promotion of improved infant and young child feeding can reduce growth faltering, although this approach falls far short of eliminating the problem. Interventions will likely need to address the complex set of underlying determinants of growth faltering, including prenatal or preconception factors. Future analyses will explore changes in feeding practices that resulted from the intervention.

Although there were more improvements in anthropometric measures in the combined water, sanitation, handwashing, and nutrition group versus active control than in the nutrition versus active control group, the differences were of little clinical or statistical significance. We conclude that combining nutrition with water, sanitation, and handwashing did not provide additional growth benefits beyond nutrition alone. Although the effect of water, sanitation, handwashing, and nutrition on mortality was not significant, the lower mortality in that group is consistent with the statistically significant effect of water, sanitation, handwashing, and nutrition on mortality in the Bangladesh trial.^9^ Pending analyses will evaluate potential differences in effects on other child health outcomes.

It is possible that the water, sanitation, and handwashing interventions delivered in this trial did not sufficiently address important transmission routes for enteric pathogens.^11^ Although the sanitation intervention included a sani-scoop and messages about preventing children from being exposed to domestic animal faeces, the emphasis was mostly on behaviours related to human faeces and might not have protected children from zoonotic pathogens.^27^ Although chlorination of water has the advantage of providing residual protection against recontamination, it is not effective against protozoa such as *Giardia lamblia* and *Cryptosporidium* spp, the latter of which was identified as one of the most common causes of moderate-to-severe diarrhoea in children 0–23 months in a neighbouring part of Kenya.^28^ Other limitations of this trial include the inability to mask the interventions; the absence of observable indicators of actual behaviour for the handwashing, sanitation, and nutrition interventions; lower adherence to the water and hygiene interventions during the second year of the trial than in the first year; and the use of a compound-level sanitation intervention, as opposed to community-level. Because masking was not possible, we focused on objective, observable indicators whenever possible rather than self-reported behaviours, recognising that the availability of a latrine or handwashing station stocked with water and soap does not necessarily imply that the materials were used. Despite an intensive design process that drew heavily on best practices in behaviour change, incorporation of lessons learned from the pilot randomised controlled trial, thorough verification of availability of the intervention materials, and periodic monitoring of indicators of recommended behaviours, adherence to the water and handwashing interventions appeared to reduce sharply in the last months of the trial. The waning intensity of promotion activities after a reduction in the stipend given to the health promoters could at least partly explain the drop in adherence. Finally, by contrast with water, handwashing, and nutrition interventions that directly benefit households that adhere to the intervention, a sanitation intervention in only a subset of compounds might not be sufficient to protect against exposure to faecal contamination in the environment that originates from other compounds in the community. We decided, however, to deliver compound-level interventions based on evidence that child exposure to enteric pathogens during the first 2 years of life occurs predominantly within the household compound.^29^ Because environmental contamination and disease transmission pathways could be different in densely populated contexts, similar studies in urban areas would complement this rural trial.

Additional outcome measures collected in this trial will help to elucidate potential mechanisms for the observed effects, including indicators of environmental contamination, environmental enteric dysfunction, anaemia, enteric parasite infection, and child development. Molecular measurement of infections in the laboratory with stored stool specimens collected as part of this trial offer an opportunity for unbiased indicators of pathogen burden.

More intensive promotion and higher adherence could have resulted in larger effects than those reported, but our findings are relevant for large-scale programmes that struggle to achieve adherence rates as high as those of efficacy studies. The potential for water, sanitation, hygiene, and nutrition interventions to reduce diarrhoea and improve growth might be highly context-dependent. In our rural setting, water was plentiful but rarely available on premises, susceptible to contamination at the source and in storage, and rarely treated despite introduction of a nearly-universal filter distribution programme;^30^ unimproved latrine coverage was high and there was a culture of using sanitation facilities for defecation by human beings, but there was probably persistent exposure to animal faeces; handwashing was not a common practice; breastfeeding was common, but exclusive breastfeeding was not, and most people had enough food, but not a diverse diet; diarrhoea prevalence was high; and many children had low length-for-age *Z* score, but not weight-for-length *Z* score. Our findings call into question the ability of large-scale water, sanitation, and handwashing interventions to reduce diarrhoea or improve growth. Our results suggest that integrated water, sanitation, and handwashing and nutrition programmes are no more effective than nutrition programmes at reducing diarrhoea or improving growth, and that nutritional interventions that include counselling and LNS can modestly reduce growth faltering, but fall short of eliminating it, even when LNS adherence is high.
