# Supplementary material for: Effects of water quality, sanitation, handwashing, and nutritional interventions on diarrhoea and child growth in rural Kenya: a cluster-randomised controlled trial
Source: Lancet Glob Health. 2018 Jan 29;6(3):e316–29. doi: 10.1016/S2214-109X(18)30005-6 (PMC5809717; doi:10.1016/S2214-109X(18)30005-6)
Supplement: Supplementary appendix [file mmc1.pdf]

THE LANCET
Global Health

Supplementary appendix

This appendix formed part of the original submission and has been peer reviewed. We post it as supplied by the authors.

Supplement to: Null C, Stewart CP, Pickering AJ, et al. Effects of water quality, sanitation, handwashing, and nutritional interventions on diarrhoea and child growth in rural Kenya: a cluster-randomised controlled trial. *Lancet Glob Health* 2018; published online Jan 29. http://dx.doi.org/10.1016/S2214-109X(18)30005-6.

Supplementary Information for WASH Benefits Primary Outcomes

TABLE OF CONTENTS

Additional information about the interventions…………………………………………………………….………..1

Details on measurements and outcome definitions…………………………………………………….………..1

CONSORT abstract checklist……………………………………………………………………………………………4

CONSORT manuscript checklist………………………………………………………………………..……….…...6

Figure S1: Location of study clusters…………….……………………………………………………………..…11

Figure S2: Box-and-whisker plots of child age in months by study arm at year 1 and year 2…..12

Figure S3: Intervention effects on diarrhea prevalence 1 year after intervention………….………..12

Figure S4: Diarrhoea prevalence by calendar month…………………………………………………………13

Figure S5: Intervention effects on length for age Z-scores after 1 year of intervention…………..14

Table S1: Child growth Z-scores after 1 year of intervention………………………………………………..15

Table S2: Child growth Z-scores after 2 years of intervention – single and combined arms vs. control arm – unadjusted and adjusted analyses……………………………………………........16

Table S3: Child growth Z-scores after 2 years of intervention – nutrition + WSH arm vs. single nutrition and WSH arms – unadjusted and adjusted analyses…………………….….........17

Table S4: Proportion of children stunted, severely stunted, wasted, and underweight after 2 years of intervention – single and combined arms vs. control arm – unadjusted and adjusted analyses ……………………………………….…………………………………..……..........18

Table S5: Proportion of children stunted, severely stunted, wasted, and underweight after 2 years of intervention – nutrition + WSH arm vs. single nutrition and WSH arms – unadjusted and adjusted analyses ………………………………………………………………..........19

Table S6: Mean outcomes among children in control clusters stratified by quantile of intervention household density within 2 km……………………………………………..……….20

Table S7: All-cause mortality among index children 2 years after intervention……………..……….21

Table S8: Intervention adherence at scale…………………………………………………..………………..……22

Additional information about the interventions

Chlorine dispensers and bottled chlorine were both 1.25% solution for a dose of 2mg/L. Each 1L bottle contained enough doses to treat 333 twenty-liter jerry-cans (for a family of five, consuming 2L of water per person per day, the bottle would last almost a year). Locations for chlorine dispensers were based on the list of sources study participants reported using for drinking water collection at baseline.

Landowners of communal water sources consented to chlorine dispenser installation, and compound heads agreed to latrine upgrades and construction. The pregnant women enrolled in the trial consented to receive all other intervention materials (including LNS).

Chlorine dispensers were installed by IPA before the community meetings. Promoters distributed bottled chlorine, potties, and faeces-removal tools within the two weeks following the community meeting. Handwashing stations were installed by IPA on a similar timeframe. Latrine upgrades and construction were subcontracted and took slightly longer, but were also complete within a few weeks. All handwashing stations and latrines were inspected by IPA staff to ensure construction was complete and met quality standards. LNS was introduced to each child on his or her six-month birthday.

To prevent the potties and sani-scoops from becoming a source of faecal contamination in the compound, caregivers were instructed to wash the potty and sani-scoop with water and soap after using the tools to move feces into the latrine and then to store the tools out of the reach of children so that they did not become an exposure risk (see the visual aids provided to participants: https://osf.io/9r4kg/ for potties and https://osf.io/mz2c6/ for sani-scoops). Health promoters emphasized the importance of making sure children understood that the potty and sani-scoop were not toys and should be used only for defecation / faeces removal. Health promoters coached caregivers to teach children to alert an adult when they needed to use the potty.

Caregivers of children who moved from their original home during the course of the study were encouraged to take their intervention materials with them to the new location. Relocated households that were still accessible via a public transit trip costing no more than 100 Kenyan shillings continued to be visited by their health promoter quarterly and LNS was delivered to them. The study did not provide latrine upgrades at the new residence.

Details on measurements and outcome definitions

Measures of intervention compliance were observed by field enumerators at the compound. One to two days later, caregivers brought the study children to a central location in the cluster for the health and anthropometry measures. Study staff attempted to schedule visits with children who were reported to still be living in the village but who did not attend the session at the central location. A separate team of enumerators and anthropometrics administered an abbreviated survey to a subset of children who were no longer living in their home village at the time of the Y2 data collection; this team also made multiple scheduled visits and followed study children as far as Mombasa. Ultimately between 6-9% of Y2 observations per arm were collected by the roving data collection team.

Caregivers reported whether the child had three or more stools in 24 hours, watery or soft stool, or any stool with blood on the day of the survey, the previous day, two days prior, or any other time in the past seven days. Diarrhea prevalence in the past seven days was defined by the combination of 3 or more watery stools in 24 hours, or by a single episode of blood in the stool.

Child ages were recorded from the clinic card or health booklet, any other birth record such as a baptismal card, or the caregiver's memory if no record was available. Date of birth was based on a clinic card, health booklet, or baptismal card for over 80% of children included in the analysis. The exact same date of birth was recorded on at least two data collection occasions for over 90% of children included in the analysis. Age ranges were wider than expected (from 2-18 months at Y1 when 9-15 months was expected and from 16-31 months at Y2 when 21-27 months was expected) due to a combination of variation in timing across arms (in particular, political unrest after the first 500 participants were enrolled shifted their timeline slightly) and the fact that we did not conduct pregnancy tests as part of the enrollment process.

Paired anthropometrists collected measurements following standard protocols[1,2] including: length with a stadiometer accurate to 0.1 cm (recumbent for children <24 months and standing for children ≥24 months), head circumference and MUAC with a non-stretchable tape (Weigh and Measure, Olney MD), and weight (without clothing) in the caregiver's arms using the tare function of the Seca 874 scale. The analysis used the median of triplicate measurements. Anthropometrists were required to pass a practical exam to demonstrate accuracy and reliability relative to an expert at the end of a three-week training before Y1 data collection and after a week-long re-training before Y2.

Exclusion ranges for outliers in anthropometry data were based on cutoff values recommended by the WHO Growth Standards (http://www.who.int/childgrowth/software/en/)
Length for age z-score: LAZ<-6 or LAZ>6
Weight for age z-score: WAZ<-6 or WAZ>5
Weight for length z-score: WLZ<-5 or WLZ>5
Head circumference for age z-score: HCZ<-5 or HCZ>5

Definitions of intervention adherence measures (figure 2):
Visited by promoter in past month - respondent report. Note that is indicator does not reflect that 27% of households reported being visited by their promoter between one and two months prior to the Y2 survey. On a related note, monitoring data from over 2300 participants surveyed in month 19 suggest that between 60% (in W and H arms) and 90% (in WSHN arms) had been visited by their promoter in the past month; much higher than the rates observed several months later at Y2.

Stored drinking water has detectable free chlorine - respondent reported the water was treated with chlorine (either from dispenser, bottle, PUR, aquatabs, or from the source) and the Hach color wheel test read >0 mg/L; households that did not report the water was treated with water are counted as not having free chlorine and households with no stored water in the home or who did not consent to the test are set to missing

Access to an improved latrine - respondent reported that their household had access to a toilet and field enumerators observed that it had a plastic or cement slab or a ventilation pipe; households who reported having access to a toilet but did not consent to letting field enumerators observe it are set to missing

Child faeces safely disposed - caregiver reported that the child's most recent defecation was directly into the latrine, into a diaper and put in the latrine, or into the potty and put in the latrine

Handwashing location has water and soap - the field enumerator observed water and some form of soap available at the primary or secondary handwashing location identified by the respondent

LNS compliance - respondent's report of how many sachets the child had consumed in the past week divided by 14

References

1. Cogill B. Anthropometric indicators measurement guide. Food and Nutrition Technical Assistance (FANTA) Project, FHI 360, Washington DC, 2003.

2. de Onis M, Onyango AW, Van den Broeck J, Chumlea WC, Martorell R. Measurement and standardization protocols for anthropometry used in the construction of a new international growth reference. Food and Nutrition Bulletin 2004; 25(1 Suppl): S27-36.

CONSORT Abstract Checklist

Item	Description	Response
**Title**	Identification of the study as randomised	Included in the title
**Authors**	Contact details for the corresponding author	Included on page 1
**Trial design**	Description of the trial design	Included in title and methods
**Methods**		
Participants	Eligibility criteria for participants in the settings where the data were collected	The methods section specifies pregnant women in rural Kenya.
Interventions	Interventions intended for each group	The six interventions are specified in the methods section.
Objective	Specific objective or hypothesis	Stated explicitly as the last sentence of the background section.
Outcome	Clearly defined primary outcome for this report	Stated in the third to last sentence of the methods section.
Randomisation	How participants were allocated to interventions	Stated in the second sentence of the methods section.
Blinding	Whether or not participants, caregivers and those assessing the outcomes were blinded to group assignment	Stated in the second to last sentence of the methods section.
**Results**		
Numbers randomized	Number of participants randomised to each group	Included in the revised summary.
Recruitment	Trial status	Implicit in journal publication that this is the final report.
Numbers analyzed	Number of participants analyzed in each group	Stated in the second sentence of the findings section. Because not all children had data on both primary outcomes, it would be too cumbersome to report the number analyzed for each outcome in each of the 8 arms of the trial; we have reported the total number analyzed and the share of living children who were measured.
Outcome	For the primary outcome, a result for each group and the estimated effect size and its precision	Included for each group for which a statistically significant effect was found; for effects that were not statistically different than zero, we have only stated that there was no effect and not included the precision of the estimates. Inclusion of

		these negative results would further lengthen the abstract and not change the message. If the editors prefer, we can lengthen the abstract and include them.
Harms	Important adverse event or side effects	None noted so we judge this as not worth the space to report. We are willing to add a sentence if the editors request it.
**Conclusions**	General interpretation of the results	Included.
**Trial registration**	Registration number and name of trial register	State in the first sentence of the methods section.
**Funding**	Source of funding	Included.

CONSORT Manuscript Checklist

Item	Description	Reported in Section
**Title and Abstract**		
1a	Identification as a randomised trial in the title; Identification as a cluster randomised trial in the title	Title
1b	Structured summary of trial design, methods, results, and conclusions	Summary
**Introduction**		
Background and Objectives		
2a	Scientific background and explanation of rationale; Rationale for using a cluster design	The study rationale is described in the introduction.<br><br>We have added additional text to the methods section to clarify, "We used a cluster design to facilitate the logistics of the behavior change component of the interventions."
2b	Specific objectives or hypotheses; Whether objectives pertain to the cluster level, the individual participant level, or both	The study's three hypotheses (entailing 11 comparisons) are stated in the second paragraph of the study design sub-section of the methods section. We have added additional text to the methods section to clarify, "We hypothesized that the interventions would be effective at the individual level."
**Methods**		
Trial Design		
3a	Description of trial design (such as parallel, factorial) including allocation ratio; Definition of cluster and description of how the design features apply to the clusters	Included in Methods under Study design, Participants, Randomisation and masking
3b	Important changes to methods after trial commencement (such as eligibility criteria), with reasons	Not applicable
Participants		
4a	Eligibility criteria for participants; Eligibility criteria for clusters	Methods (Participants)
4b	Settings and locations where the data were collected	Methods (Study design and Procedures)

Item	Description	Reported in Section
Interventions		
5	The interventions for each group with sufficient details to allow replication, including how and when they were actually administered; Whether interventions pertain to the cluster level, the individual participant level, or both	Methods (Procedures)
Outcomes		
6a	Completely defined pre-specified primary and secondary outcome measures, including how and when they were assessed; Whether outcome measures pertain to the cluster level, the individual participant level, or both	Methods (Procedures; Outcomes)<br><br>We concede that we do not list all of the primary and secondary outcomes, though we provide a link both to the design paper and to the analysis plan that presents this in exhaustive detail.
6b	Any changes to trial outcomes after the trial commenced, with reasons	Not applicable
Sample Size		
7a	How sample size was determined; Method of calculation, number of cluster(s) (and whether equal or unequal cluster sizes are assumed), cluster size, a coefficient of intracluster correlation (ICC or k), and an indication of its uncertainty	Methods (Statistical analysis)
7b	When applicable, explanation of any interim analyses and stopping guidelines	Not applicable
**Randomisation**		
Sequence Generation		
8a	Method used to generate the random allocation sequence	Methods (Randomisation and masking)
8b	Type of randomisation; details of any restriction (such as blocking and block size); Details of stratification or matching if used	Methods (Randomisation and masking)
Allocation Concealment Mechanism		
9	Mechanism used to implement the random allocation sequence (such as sequentially numbered containers), describing any steps taken to conceal	Methods (Randomisation and masking)

Item	Description	Reported in Section
	the sequence until interventions were assigned; Specification that allocation was based on clusters rather than individuals and whether allocation concealment (if any) was at the cluster level, the individual participant level, or both	
Implementation		
10a	Who generated the random allocation sequence, who enrolled clusters, and who assigned clusters to interventions	Methods (Participants, Randomisation and masking)
10b	Mechanism by which individual participants were included in clusters for the purposes of the trial (such as complete enumeration, random sampling)	Methods (Participants)
10c	From whom consent was sought (representatives of the cluster, or individual cluster members, or both) and whether consent was sought before or after randomisation	Methods (Participants)
Blinding		
11a	If done, who was blinded after assignment to interventions (for example, participants, care providers, those assessing outcomes) and how	Methods (Randomisation and masking)
11b	If relevant, description of the similarity of interventions	Not applicable
Statistical Methods		
12a	Statistical methods used to compare groups for primary and secondary outcomes; How clustering was taken into account	Methods (Statistical analysis)
12b	Methods for additional analyses, such as subgroup analyses and adjusted analyses	Methods (Statistical analyses)
**Results**		
Participant Flow		
13a	For each group, the numbers of participants/clusters who were randomly assigned, received intended	Results; Figure 1

Item	Description	Reported in Section
	treatment, and were analyzed for the primary outcome	
13b	For each group, losses and exclusions after randomization, together with reasons, for both clusters and individual cluster members	Results; Figure 1
Recruitment		
14a	Dates defining the periods of recruitment and follow-up	Results
14b	Why the trial ended or was stopped	Not applicable
Baseline Data		
15	A table showing baseline demographic and clinical characteristics for each group; Baseline characteristics for the individual and cluster levels as applicable for each group	Table 1
Numbers Analysed		
16	For each group, number of participants/clusters (denominator) included in each analysis and whether the analysis was by the original assigned groups	Methods (Statistical analyses); Figure 1
Outcomes and Estimation		
17a	For each primary and secondary outcome, results for each group, and the estimated effect size and its precision (such as 95% confidence interval); Results at the individual and cluster levels as applicable and a coefficient of intracluster correlation (ICC or k) for each primary outcome	Results Figure 2, Figure 3, Table 3, Table 4.
17b	For binary outcome, presentation of both absolute and relative effect sizes is recommended	Results, Figure 2 and Table S1
Ancillary Analyses		
18	Results of any other analyses performed, including subgroup analyses and adjusted analyses, distinguishing pre-specified from exploratory	Methods (Statistical analyses); Results; Supplemental Information
Harms		

Item	Description	Reported in Section
19	All important harms or unintended effects in each group	Not applicable
**Discussion**		
Limitations		
20	Trial limitations, addressing sources of potential bias, imprecision and, if relevant, multiplicity of analyses	Discussion
Generalisability		
21	Generalisability (external validity, applicability) of the trial findings; Generalisability to clusters and/or individual participants (as relevant)	Discussion
Interpretation		
22	Interpretation consistent with results, balancing benefits and harms, and considering other relevant evidence	Discussion
**Other Information**		
Registration		
23	Registration number and name of trial registry	Methods (Statistical analyses)
Protocol		
24	Where the full trial protocol can be accessed, if available	Methods (Statistical analyses)
Funding		
25	Sources of funding and other support (such as supply of drugs), role of funders	Summary (Funding); Methods (Role of the funding source)

Figure S1: Location of study clusters

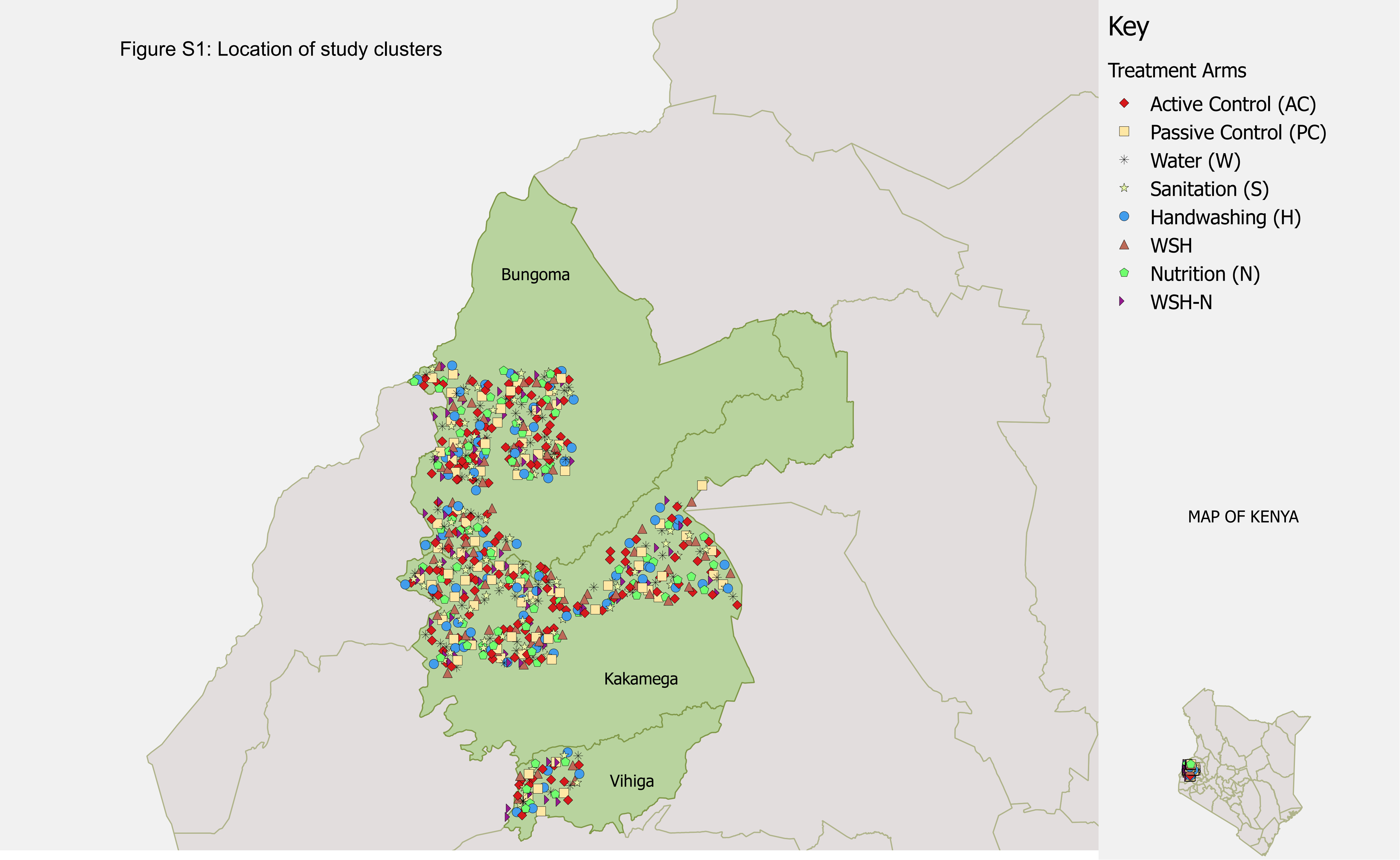

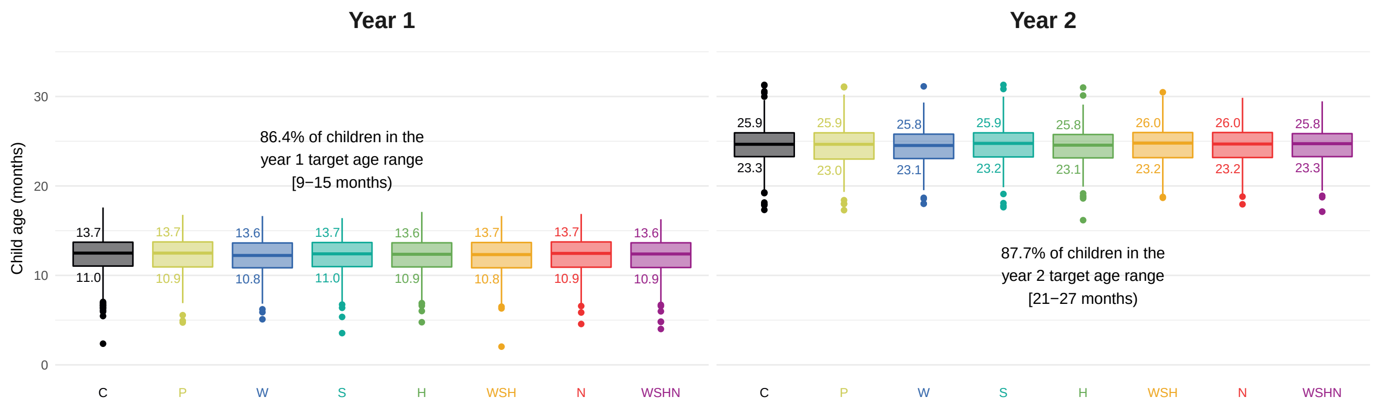

Figure S2: Box-and-whisker plots of child age in months by study arm at year 1 and year 2, 12 and 24 months after delivery of the interventions. The line across the box denotes the median age. The bottom and top of the boxes, and printed numbers, are the 25th and 75th percentiles respectively. The whiskers mark 1.5 times the inter-quartile range, the difference between the 75th and 25th percentiles, and points mark outliers beyond the whiskers. C: active control; P: passive control; W: water treatment; S: sanitation; H: handwashing; WSH: combined water, sanitation, handwashing; N: nutrition; WSHN: combined water, sanitation, handwashing, nutrition

Prevalence Ratio (95% CI)	Control	Passive Control	Water	Sanitation	Handwashing	Combined WSH	Nutrition	Combined Nutrition+WSH
Intervention v. Control	ref	0.95 (0.82, 1.10)	1.01 (0.87, 1.16)	0.93 (0.80, 1.08)	0.91 (0.78, 1.06)	0.91 (0.78, 1.05)	0.96 (0.82, 1.12)	0.99 (0.86, 1.14)
WSH v. Water			ref			0.88 (0.74, 1.05)		
WSH v. Sanitation				ref		0.99 (0.83, 1.19)		
WSH v. Handwashing					ref	1.00 (0.83, 1.20)		

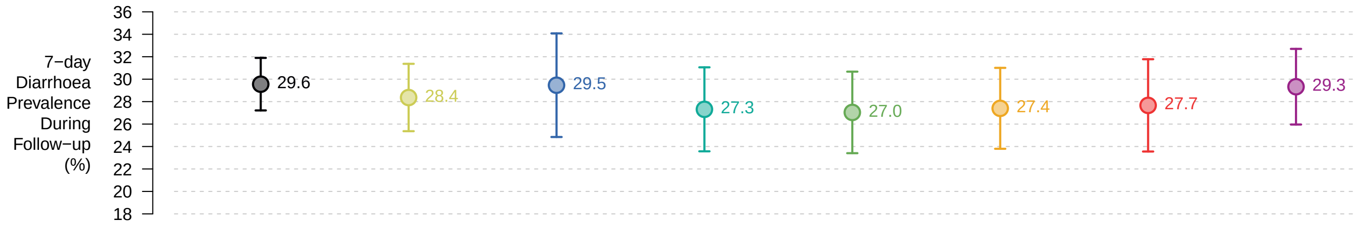

Figure S3: Intervention effects on diarrhoea prevalence 1 year after intervention. Seven day diarrhoea prevalence was measured twice during the study, at 1 and 2 years after the delivery of interventions. Mantel-Haenszel prevalence ratios (stratified by matched pair) and their 95% confidence intervals (CI) compare the passive control arm and each intervention arm against the active control arm, as well as the combined water, sanitation, and handwashing (WSH) package against single water, sanitation, or handwashing interventions.

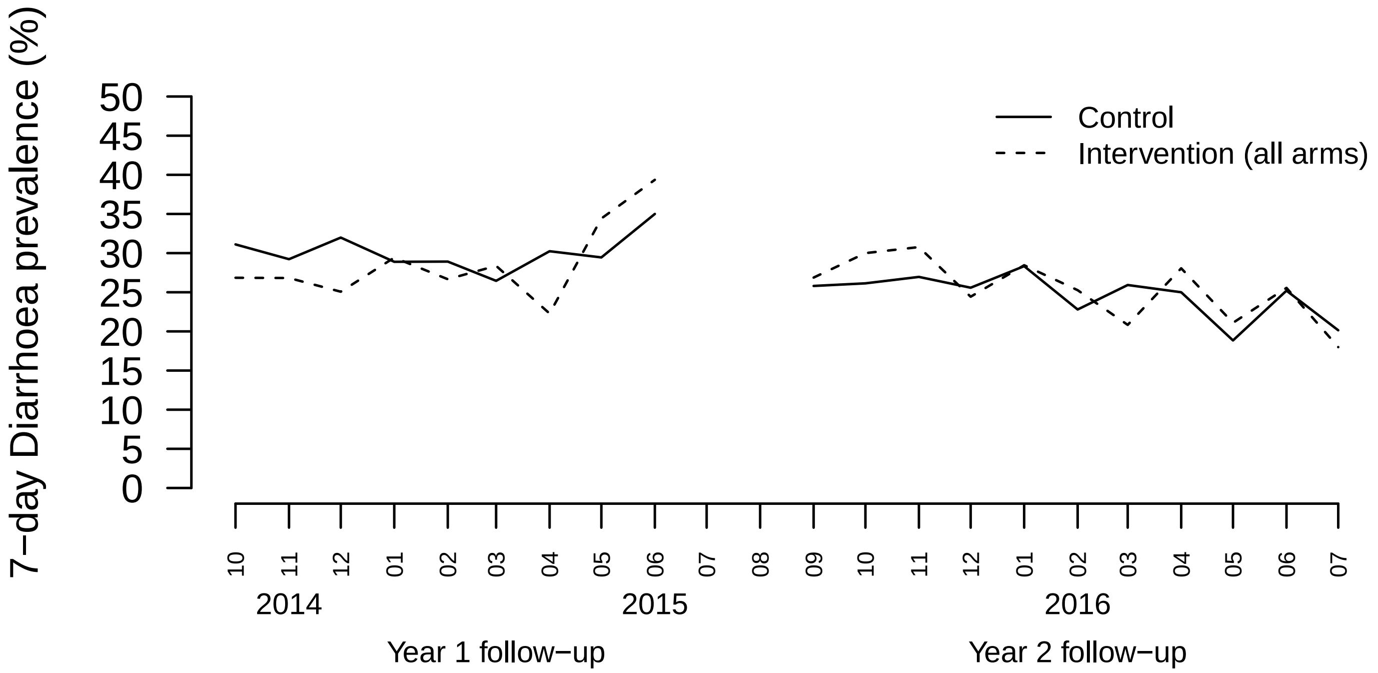

Figure S4: Diarrhoea prevalence by calendar month. Individual children were measured only once at each round of follow-up, but due to the large study size each follow-up measurement spanned approximately one calendar year. Control and intervention clusters were geographically matched and matched clusters were measured concurrently. All intervention arms had similar prevalence during follow-up (Fig 3), and were combined into a single data series in this figure to have sufficient observations to estimate monthly prevalence. The control data series includes on average 208 observations per month (range: 100, 309) and the intervention data series includes on average 400 observations per month (range: 188, 570).

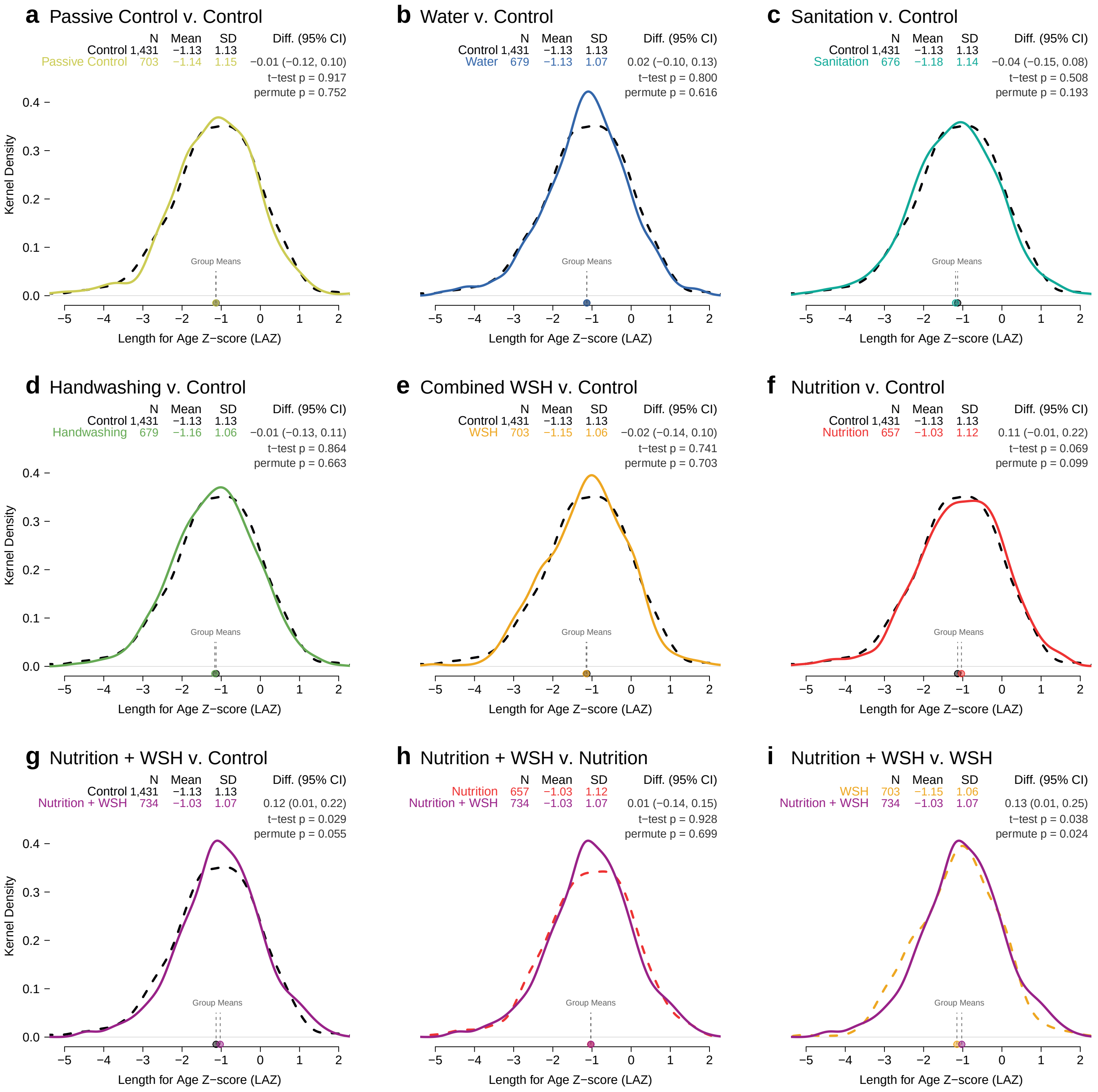

Figure S5: Intervention effects on length for age Z-scores (LAZ) among 6,262 children after 1 year of intervention. Kernel density plots summarize the distribution of LAZ among index children who were born into the study and were between 2-18 months (median = 12 months) at the time of measurement. In each panel, a dashed line illustrates the comparison group distribution and a solid line illustrates the active comparator distribution. a-g, passive control and each active intervention arm compared against the double-sized active control arm; h,i, combined water, sanitation, handwashing (WSH) plus nutrition compared against either nutrition alone (h) or WSH alone (i). t-test p-values test whether differences in group means are different from zero; permutation p-values test the strong null hypothesis of no difference between groups using a Wilcoxon signed-rank test statistic.

Table S1: Child growth Z-scores after 1 year of intervention

Outcome, Arm	N	Mean	SD	Difference vs. Control (95%)	Difference vs. Nutrition (95%)	Difference vs. WSH (95%)
**Weight-for-age Z-score***						
Control	1441	-0.38	1.11			
Passive Control	709	-0.40	1.14	0.00 (-0.11, 0.11)		
Water	684	-0.35	1.16	0.03 (-0.10, 0.16)		
Sanitation	679	-0.46	1.14	-0.06 (-0.17, 0.05)		
Handwashing	685	-0.38	1.12	0.00 (-0.12, 0.11)		
Water + Sanitation + Handwashing (WSH)	709	-0.39	1.09	0.01 (-0.11, 0.13)		
Nutrition	662	-0.24	1.09	0.16 (0.03, 0.28)		
Nutrition + WSH	738	-0.20	1.06	0.19 (0.08, 0.30)	0.04 (-0.09, 0.18)	0.18 (0.05, 0.31)
**Weight-for-length Z-score***						
Control	1430	0.25	1.03			
Passive Control	704	0.22	1.08	-0.02 (-0.12, 0.08)		
Water	679	0.30	1.11	0.02 (-0.10, 0.15)		
Sanitation	678	0.16	1.05	-0.07 (-0.18, 0.03)		
Handwashing	679	0.27	1.07	-0.01 (-0.12, 0.10)		
Water + Sanitation + Handwashing (WSH)	704	0.26	1.06	0.03 (-0.08, 0.14)		
Nutrition	657	0.36	1.02	0.13 (0.01, 0.25)		
Nutrition + WSH	735	0.40	1.00	0.16 (0.05, 0.26)	0.04 (-0.08, 0.16)	0.13 (0.00, 0.25)
**Head circumference-for-age Z-score***						
Control	1441	-0.25	1.02			
Passive Control	707	-0.30	1.03	-0.04 (-0.14, 0.06)		
Water	682	-0.23	1.02	0.04 (-0.06, 0.15)		
Sanitation	674	-0.32	1.07	-0.06 (-0.16, 0.05)		
Handwashing	683	-0.26	1.00	-0.02 (-0.13, 0.10)		
Water + Sanitation + Handwashing (WSH)	709	-0.30	0.99	-0.05 (-0.16, 0.07)		
Nutrition	661	-0.20	1.03	0.05 (-0.06, 0.16)		
Nutrition + WSH	735	-0.19	0.99	0.07 (-0.03, 0.16)	-0.01 (-0.13, 0.11)	0.09 (-0.03, 0.21)

The median child age was 1.03 years (IQR: 0.91-1.14).
* Pre-specified secondary outcome

Table S2: Child growth Z-scores after 2 years of intervention - single and combined arms vs. control arm - Unadjusted and adjusted analyses

		Difference from Control (95%)	
Arm	Mean	Unadjusted	Adjusted*
**Length-for-age Z-score**			
Control	-1.54		
Passive Control	-1.56	-0.02 (-0.13, 0.09)	-0.01 (-0.11, 0.09)
Water	-1.58	-0.04 (-0.15, 0.08)	-0.05 (-0.14, 0.05)
Sanitation	-1.61	-0.06 (-0.18, 0.05)	-0.05 (-0.15, 0.06)
Handwashing	-1.60	-0.04 (-0.16, 0.07)	-0.02 (-0.12, 0.08)
Water + Sanitation + Handwashing (WSH)	-1.59	-0.03 (-0.14, 0.08)	-0.04 (-0.14, 0.06)
Nutrition	-1.44	0.13 (0.01, 0.25)	0.10 (-0.01, 0.21)
Nutrition + WSH	-1.39	0.16 (0.05, 0.27)	0.18 (0.07, 0.29)
**Weight-for-age Z-score**†			
Control	-0.72		
Passive Control	-0.76	-0.04 (-0.13, 0.05)	-0.04 (-0.13, 0.05)
Water	-0.73	0.00 (-0.10, 0.10)	0.00 (-0.09, 0.09)
Sanitation	-0.80	-0.07 (-0.19, 0.04)	-0.06 (-0.16, 0.04)
Handwashing	-0.77	-0.05 (-0.15, 0.05)	-0.03 (-0.11, 0.05)
Water + Sanitation + Handwashing (WSH)	-0.77	-0.02 (-0.12, 0.08)	-0.03 (-0.12, 0.06)
Nutrition	-0.65	0.11 (0.00, 0.21)	0.07 (-0.03, 0.17)
Nutrition + WSH	-0.60	0.14 (0.04, 0.25)	0.14 (0.04, 0.23)
**Weight-for-length Z-score**†			
Control	0.11		
Passive Control	0.08	-0.04 (-0.13, 0.05)	-0.05 (-0.13, 0.04)
Water	0.14	0.04 (-0.06, 0.13)	0.04 (-0.06, 0.13)
Sanitation	0.05	-0.05 (-0.14, 0.05)	-0.05 (-0.14, 0.04)
Handwashing	0.09	-0.02 (-0.11, 0.06)	-0.01 (-0.08, 0.06)
Water + Sanitation + Handwashing (WSH)	0.08	-0.02 (-0.10, 0.07)	-0.02 (-0.09, 0.05)
Nutrition	0.14	0.04 (-0.05, 0.14)	0.02 (-0.07, 0.11)
Nutrition + WSH	0.18	0.09 (0.00, 0.19)	0.07 (-0.02, 0.16)
**Head circumference-for-age Z-score**†			
Control	-0.27		
Passive Control	-0.27	0.00 (-0.10, 0.10)	-0.05 (-0.14, 0.04)
Water	-0.27	0.02 (-0.08, 0.12)	-0.03 (-0.13, 0.06)
Sanitation	-0.27	0.01 (-0.09, 0.11)	0.01 (-0.09, 0.10)
Handwashing	-0.29	0.00 (-0.10, 0.10)	-0.01 (-0.09, 0.08)
Water + Sanitation + Handwashing (WSH)	-0.30	-0.03 (-0.12, 0.06)	-0.05 (-0.14, 0.04)
Nutrition	-0.23	0.05 (-0.05, 0.15)	0.01 (-0.09, 0.11)
Nutrition + WSH	-0.22	0.05 (-0.04, 0.15)	0.08 (0.00, 0.16)

The median child age was 2.05 years (IQR: 1.93-2.16).

* Adjusted for pre-specified covariates using targeted maximum likelihood estimation with data-adaptive model selection [Arnold 2013, Balzer 2016]: Field staff who collected data, month of measurement, household food insecurity, child age, child sex, mother's age, mothers height, mothers education level, number of children < 18 years in the household, number of individuals living in the compound, distance in minutes to the primary water source, household roof, floor, wall materials, household assets.

† Pre-specified secondary outcome

Table S3: Child growth Z-scores after 2 years of intervention - Nutrition + WSH arm vs. single Nutrition and WSH arms - Unadjusted and adjusted analyses

		Nutrition + WSH minus single arms (95% CI)	
Arm	Mean	Unadjusted	Adjusted*
**Length-for-age Z-score**			
Nutrition + WSH	-1.39		
Nutrition	-1.44	0.04 (-0.11, 0.19)	0.04 (-0.09, 0.17)
WSH	-1.59	0.19 (0.08, 0.31)	0.20 (0.10, 0.31)
**Weight-for-age Z-score**†			
Nutrition + WSH	-0.60		
Nutrition	-0.65	0.04 (-0.07, 0.15)	0.05 (-0.06, 0.15)
WSH	-0.77	0.17 (0.05, 0.30)	0.18 (0.07, 0.30)
**Weight-for-length Z-score**†			
Nutrition + WSH	0.18		
Nutrition	0.14	0.04 (-0.05, 0.13)	0.04 (-0.06, 0.14)
WSH	0.08	0.12 (0.00, 0.23)	0.11 (0.00, 0.21)
**Head circumference-for-age Z-score**†			
Nutrition + WSH	-0.22		
Nutrition	-0.23	-0.02 (-0.14, 0.10)	0.03 (-0.08, 0.13)
WSH	-0.30	0.08 (-0.05, 0.20)	0.12 (0.00, 0.23)

The median child age was 2.05 years (IQR: 1.93-2.16).

* Adjusted for pre-specified covariates using targeted maximum likelihood estimation with data-adaptive model selection [Arnold 2013, Balzer 2016]: Field staff who collected data, month of measurement, household food insecurity, child age, child sex, mother's age, mothers height, mothers education level, number of children < 18 years in the household, number of individuals living in the compound, distance in minutes to the primary water source, household roof, floor, wall materials, household assets.

† Pre-specified secondary outcome

Table S4: Proportion of children stunted, severely stunted, wasted and underweight after 2 years of intervention - single and combined arms vs. control arm - Unadjusted and adjusted analyses

		Difference from Control (95%)	
Outcome, Arm	Mean	Unadjusted*	Adjusted†
**Stunting‡**			
Control	31.5%		
Passive Control	31.1%	-1.7 (-5.9, 2.5)	-1.3 (-6.2, 3.5)
Water	32.4%	0.1 (-4.2, 4.3)	0.8 (-3.3, 5.0)
Sanitation	34.5%	2.3 (-2.0, 6.6)	1.7 (-3.0, 6.4)
Handwashing	33.6%	0.8 (-3.5, 5.1)	0.3 (-3.7, 4.3)
Water + Sanitation + Handwashing (WSH)	32.8%	1.3 (-3.0, 5.6)	0.0 (-4.6, 4.7)
Nutrition	28.9%	-3.2 (-7.5, 1.1)	-2.2 (-7.3, 2.9)
Nutrition + WSH	26.7%	-5.4 (-9.4, -1.4)	-6.2 (-10.8, -1.6)
**Severe stunting§**			
Control	9.3%		
Passive Control	8.7%	-0.8 (-3.3, 1.8)	-0.5 (-3.1, 2.0)
Water	9.6%	-0.5 (-3.2, 2.2)	0.5 (-2.4, 3.3)
Sanitation	10.4%	1.0 (-1.8, 3.7)	1.2 (-2.0, 4.3)
Handwashing	8.4%	-1.1 (-3.7, 1.5)	-0.3 (-2.9, 2.3)
Water + Sanitation + Handwashing (WSH)	9.0%	0.2 (-2.4, 2.8)	0.4 (-2.2, 3.1)
Nutrition	7.9%	-1.6 (-4.2, 1.0)	-1.1 (-3.9, 1.7)
Nutrition + WSH	7.2%	-2.7 (-5.1, -0.2)	-2.2 (-4.6, 0.1)
**Wasting§**			
Control	1.4%		
Passive Control	1.4%	0.0 (-1.1, 1.1)	0.2 (-0.9, 1.2)
Water	1.3%	-0.2 (-1.3, 0.8)	-0.3 (-1.4, 0.8)
Sanitation	2.6%	1.1 (-0.3, 2.4)	1.1 (-0.1, 2.3)
Handwashing	0.9%	-0.5 (-1.5, 0.4)	-0.3 (-1.3, 0.7)
Water + Sanitation + Handwashing (WSH)	1.4%	0.2 (-0.9, 1.2)	0.0 (-0.9, 0.9)
Nutrition	1.2%	-0.3 (-1.3, 0.8)	-0.3 (-1.3, 0.7)
Nutrition + WSH	1.4%	-0.1 (-1.2, 1.0)	0.1 (-1.2, 1.5)
**Underweight§**			
Control	9.6%		
Passive Control	9.7%	-0.4 (-3.0, 2.2)	0.2 (-2.4, 2.8)
Water	10.5%	-0.1 (-2.8, 2.7)	0.5 (-2.0, 3.1)
Sanitation	11.6%	1.6 (-1.2, 4.4)	1.7 (-1.7, 5.1)
Handwashing	10.1%	0.5 (-2.2, 3.3)	0.0 (-2.7, 2.8)
Water + Sanitation + Handwashing (WSH)	9.9%	0.5 (-2.3, 3.2)	0.3 (-2.4, 2.9)
Nutrition	8.5%	-1.2 (-3.9, 1.5)	-0.4 (-3.5, 2.7)
Nutrition + WSH	6.8%	-3.0 (-5.4, -0.6)	-2.4 (-5.0, 0.2)

The median child age was 2.05 years (IQR: 1.93-2.16).

* Unadjusted estimates were estimated using a pair-matched Mantel-Haenszel analysis.

† Adjusted for pre-specified covariates using targeted maximum likelihood estimation with data-adaptive model selection [Arnold 2013, Balzer 2016]: Field staff who collected data, month of measurement, household food insecurity, child age, child sex, mother's age, mothers height, mothers education level, number of children < 18 years in the household, number of individuals living in the compound, distance in minutes to the primary water source, household roof, floor, wall materials, household assets.

‡ Pre-specified secondary outcome

§ Pre-specified tertiary outcome

Table S5: Proportion of children stunted, severely stunted, wasted and underweight after 2 years of intervention - Nutrition + WSH arm vs. single Nutrition and WSH arms - Unadjusted and adjusted analyses

		Nutrition + WSH minus single arms (95% CI)	
Outcome, Arm	Mean	Unadjusted*	Adjusted†
**Stunting**‡			
Nutrition + WSH	26.7%		
Nutrition	28.9%	-2.3 (-7.1, 2.5)	-4.1 (-9.7, 1.5)
WSH	32.8%	-5.8 (-10.6, -1.0)	-5.9 (-11.2, -0.6)
**Severe stunting**§			
Nutrition + WSH	7.2%		
Nutrition	7.9%	-0.9 (-3.7, 2.0)	-0.9 (-3.7, 1.9)
WSH	9.0%	-2.7 (-5.6, 0.2)	-2.9 (-5.8, 0.0)
**Wasting**§			
Nutrition + WSH	1.4%		
Nutrition	1.2%	0.2 (-1.0, 1.4)	0.3 (-0.8, 1.5)
WSH	1.4%	0.0 (-1.2, 1.1)	-0.1 (-1.4, 1.1)
**Underweight**§			
Nutrition + WSH	6.8%		
Nutrition	8.5%	-1.8 (-4.7, 1.1)	-2.5 (-5.5, 0.6)
WSH	9.9%	-3.3 (-6.2, -0.5)	-3.0 (-5.7, -0.3)

The median child age was 2.05 years (IQR: 1.93-2.16).

* Unadjusted estimates were estimated using a pair-matched Mantel-Haenszel analysis.

† Adjusted for pre-specified covariates using targeted maximum likelihood estimation with data-adaptive model selection [Arnold 2013, Balzer 2016]: Field staff who collected data, month of measurement, household food insecurity, child age, child sex, mother's age, mothers height, mothers education level, number of children < 18 years in the household, number of individuals living in the compound, distance in minutes to the primary water source, household roof, floor, wall materials, household assets.

‡ Pre-specified secondary outcome

§ Pre-specified tertiary outcome

Table S6: Mean outcomes among children in control clusters stratified by quantile of intervention household density within 2 km

Arm	N_{20}^{a}	N_{80}^{b}	$Mean_{20}^{c}$	$Mean_{80}^{d}$	Difference[e]	P-value[f]	Adjusted P-value[g]
**Length-for-age z-score**[h]							
Water, WSH, Nutrition + WSH	308	313	-1.541	-1.556	-0.015	0.868	0.969
Sanitation, WSH, Nutrition + WSH	307	308	-1.534	-1.618	-0.084	0.347	0.302
Handwashing, WSH, Nutrition + WSH	307	307	-1.498	-1.485	0.013	0.884	0.553
Nutrition, Nutrition + WSH	308	308	-1.529	-1.434	0.096	0.285	0.252
**Diarrhea**[i]							
Water, WSH, Nutrition + WSH	581	585	0.265	0.262	-0.004	0.888	0.511
Sanitation, WSH, Nutrition + WSH	582	583	0.259	0.261	0.001	0.950	0.518
Handwashing, WSH, Nutrition + WSH	581	584	0.244	0.262	0.018	0.490	0.352
Nutrition, Nutrition + WSH	581	582	0.250	0.239	-0.011	0.691	0.525

Intervention households were grouped by different types of intervention (sanitation, handwashing, water, nutrition). In the presence of positive between-cluster spillover effects (contamination), control children in the <20th percentile would have better outcomes (higher LAZ, lower diarrhea prevalence) compared with control children in the >80th percentile.

[a] N_{20} = the number of control households for which the number of treated households within 2 km is less than or equal to the 20th percentile of the observed distribution of distance

[b] N_{80} = the number of control households for which the number of treated households within 2 km is greater than or equal to the 80th percentile of the observed distribution of distance

[c] $Mean_{20}$ = the mean outcome in control households for which the number of treated households within 2 km is less than or equal to the 20th percentile of the observed distribution of distance

[d] $Mean_{80}$ = the mean outcome in control households for which the number of treated households within 2 km is greater than or equal to the 80th percentile of the observed distribution of distance

[e] Difference = $Mean_{80}$ - $Mean_{20}$

[f] Permutation test p-value

[g] Adjusted permutation test p-value. Models adjusted for pre-specified covariates using with data-adaptive model selection [Arnold 2013]: Field staff who collected data, month of measurement, household food insecurity, child age, child sex, mother's age, mothers height, mothers education level, number of children < 18 years in the household, number of individuals living in the compound, distance in minutes to the primary water source, household roof, floor, wall materials, household assets

[h] Post-intervention year 2 measurement

[i] Post-intervention measurements in years 1 and 2 combined

Table S7: All-cause mortality among index children 2 years after intervention

Arm	N at risk	n deaths	Cumulative incidence	Risk difference (95% CI)
Control	1895	73	0.039	
Passive Control	916	41	0.045	0.006 (-0.010, 0.023)
Water	888	30	0.034	-0.003 (-0.019, 0.012)
Sanitation	874	34	0.039	0.003 (-0.013, 0.019)
Handwashing	903	48	0.053	0.013 (-0.004, 0.030)
WSH	893	44	0.049	0.009 (-0.008, 0.025)
Nutrition	835	32	0.038	-0.001 (-0.017, 0.015)
Nutrition + WSH	897	25	0.028	-0.010 (-0.024, 0.005)

Table S8: Intervention adherence at scale

Country	Intervention	Scale	Adherence	Citation
Bangladesh	The Sanitation Hygiene Education and Water Supply in Bangladesh (SHEWA-B) project aimed to improve the hygiene, sanitation and water supply through behavior change messaging delivered by community health promoters	20 million people	Handwashing with soap observed before < 3% of food-related events and after <40% of incidents of cleaning a child's anus	Huda TM, Unicomb L, Johnston RB, Halder AK, Yushuf Sharker MA, Luby SP. Interim evaluation of a large scale sanitation, hygiene and water improvement programme on childhood diarrhea and respiratory disease in rural Bangladesh. Soc Sci Med 2012; 75(4): 604-11.
Kenya, Malawi, Uganda	Dispensers for Safe Water provides chlorine dispensers for point-of-collection water treatment at communal water sources, community education, and a regular supply of chlorine	4.7 million people	50-60% of households have chlorine residual in their water during random spot-checks each month	https://www.evidenceaction.org/dispensersforsafewater#track-record
Peru	The Global Scaling Up Handwashing Project provided mass-media, capacity building, and a primary school curricula	800 districts	<30% were observed to wash hands with soap before eating, <40% after fecal contact	Galiani S, Gertler P, Orsola-Vidal A. Promoting handwashing behavior in Peru: the effect of large-scale mass media and community level interventions. The World Bank: Policy Research Working Paper 6257, November 2012.
Tanzania	The Global Scaling Up Handwashing Project provided social marketing including community-led total sanitation triggering	2.7 million people	<15% of households had a handwashing station with soap and <20% were observed to wash hands with soap after fecal contact	Briceno B, Coville A, Martinez S. Promoting handwashing and sanitation: evidence form a large-scale randomized trial in rural Tanzania. The World Bank: Policy Research Working Paper 7164, January 2015.
Vietnam	The Global Scaling Up Handwashing project provided a year of television ads and interpersonal communication activities	3 provinces	Handwashing with soap observed before <10% of food preparation events and after <25% of fecal contact events	Chase C and Do Q. Handwashing behavior change at scale: evidence from a randomized evaluation in Vietnam. The World Bank: Research Brief, 2012.
